# Structural insights into the nuclear import of ovine gammaherpesvirus 2 ORF73 LANA homologue

**DOI:** 10.1099/jgv.0.002250

**Published:** 2026-04-17

**Authors:** Babu Kanti Nath, Renate H. M. Schwab, Crystall M. D. Swarbrick, Silvia Pavan, Nazia Rahman, Brian P. McSharry, Shane R. Raidal, Daryl Ariawan, Ole Tietz, Shubhagata Das, Gualtiero Alvisi, Jade K. Forwood

**Affiliations:** 1 Gulbali Institute, Charles Sturt University, Wagga Wagga, NSW, Australia; 2 School of Agricultural Environmental and Veterinary Sciences, Faculty of Science and Health, Charles Sturt University, Wagga Wagga, NSW, Australia; 3 Department of Molecular Medicine, University of Padua, Padua, Italy; 4 School of Dentistry and Medical Sciences, Faculty of Science and Health, Charles Sturt University, Wagga Wagga, NSW, Australia; 5 Training Hub Promoting Regional Industry and Innovation in Virology and Epidemiology, Gulbali Institute, Charles Sturt University, Wagga Wagga, NSW, Australia; 6 Melbourne Veterinary School, Faculty of Science, University of Melbourne, Victoria, Australia; 7 Dementia Research Centre, Macquarie Medical School, Faculty of Medicine, Health and Human Sciences, Macquarie University, North Ryde, Sydney, NSW 2109, Australia

**Keywords:** crystallography, importins, nuclear trafficking, ovine gammaherpesvirus 2

## Abstract

Ovine gammaherpesviruses 2 (OvGHV2), a member of the *Macavirus* genus within the *Orthoherpesviridae* family, causes lymphoproliferative diseases in susceptible species, most notably sheep-associated malignant catarrhal fever. Research on OvGHV2 has been hindered by the absence of a permissive cell culture system, limiting investigations into viral replication, entry mechanisms and cell tropism. This challenge constrains progress towards understanding OvGHV2 pathogenesis and developing effective vaccines or therapeutics. We investigated the molecular mechanisms underlying OvGHV2 infection, focusing on the nuclear trafficking pathways of the latency-associated nuclear antigen (LANA), encoded by ORF73. In other gammaherpesviruses, LANA is known to mediate viral episome maintenance, chromatin tethering and latency through its nuclear localization. We identified and functionally characterized a novel bipartite nuclear localization signal (NLS) within the C-terminal region of OvGHV2 LANA and elucidated its interactions with host nuclear import receptors. Using high-resolution crystallography and quantitative binding assays, we mapped the key residues responsible for binding to importin alpha (IMP*α*) and demonstrated isoform-specific variations in binding affinity. Confocal microscopy revealed that the OvGHV2 LANA predominantly localizes to the nucleus through an IMP*α*/*β*1-dependent pathway, as mutation or inhibition of the NLS significantly reduced nuclear accumulation. Interestingly, partial nuclear localization under these conditions suggests an additional IMP*α*/*β*1-independent nuclear import mechanism. Collectively, our biochemical and structural analyses confirm that the identified NLS is essential for IMP*α*-mediated nuclear import of OvGHV2 LANA. These findings provide new insights into OvGHV2 host interactions and establish a molecular basis for developing targeted antiviral strategies against ovine gammaherpesvirus 2 infection.

## Introduction

Ovine gammaherpesvirus 2 (OvGHV2), a rhadinovirus in the subfamily *Gammaherpesvirinae*, is the causative agent of sheep-associated malignant catarrhal fever, a severe lymphoproliferative disease in ruminants [1,2]. Sheep serve as the natural reservoir of OvGHV-2, in which infection is largely subclinical; however, cross-species transmission to susceptible hosts, such as cattle, bison, deer and pigs, typically results in high fatality [3,5]. OvGHV-2 can establish both lytic and latent infections in the host species. During the lytic phase, there is active virus replication within host cells. This results in the production of new viral particles (virions) and often leads to cell destruction and tissue damage [6,7]. Lytic infection results in the development of malignant catarrhal fever (MCF)-associated pathology and clinical signs. In the latent phase, the virus enters a quiescent or dormant state, where it does not actively replicate or produce infectious virions. Latent infections can reactivate, with the virus transitioning back to the lytic phase [1,8].

Previous studies on alcelaphine herpesvirus 1 (AlHV-1) have demonstrated that the latency-associated nuclear antigen (LANA) is critical for maintenance of the viral genome and for the induction of malignant catarrhal fever [9]. Subsequent work indicated that LANA-mediated immune evasion is not required for disease pathogenesis in susceptible species; instead, the glycine–glutamate-rich repeat region was identified as being essential for immune evasion during latent infection, likely in the wildebeest natural host [10]. The ability of OvGHV-2 to establish latent infections is a key factor in its persistence within host populations. The continuing threat of OvGHV2 to the livestock industry is of major concern, especially given the absence of effective antiviral therapies or vaccines. In light of these challenges, a deeper understanding of the molecular processes governing OvGHV2 infection, particularly its nuclear trafficking pathways, is essential.

Nuclear trafficking is a complex biological function that can be generated by a spatially and temporally organized cycle of interactions between cargoes, carriers and Ran GTPase [11]. Molecules with a molecular weight below 70 kDa can passively diffuse through the nuclear pore complex (NPC) [12]. However, larger molecules, as well as those that need rapid accumulation in the nucleus, rely on an energy-dependent transport system. This process is facilitated by cellular transporters from the importin (IMP) superfamily, which recognize nuclear localization signals (NLSs) on their cargo [12,13]. Eukaryotic cells express multiple IMPs, and although their exact number differs across species, most are highly conserved [14]. The precise rules governing IMP-cargo specificity remain unclear, but each IMP appears to have distinct cargo recognition characteristics, which are only partially understood [15]. To date, four categories of NLSs have been identified, each recognized by specific IMPs. The best-characterized type is the ‘classical’ nuclear localization signal (cNLS), which consists of short sequences rich in basic amino acids such as arginine and lysine [16]. cNLSs can be either monopartite, containing a single continuous stretch of basic residues, or bipartite, where two basic sequences are separated by a short linker [17,18]. cNLSs directly interact with IMP*α*, which acts as an adapter to bridge the cargo to IMP*β*1. IMP*β*1 then facilitates the translocation of the complex through the NPC. In contrast, non-classical nuclear localization signals (ncNLSs) do not require IMPα and can bind directly to IMP*β*1 or its orthologues, including transportin-1 (IMP*β*2), Ran-binding protein 4 (importin 4), karyopherin *β*3 (importin 5) and Ran-binding protein 7 (importin 7), among others [19,21].

The directionality of nuclear transport is maintained by the differential distribution of GDP and GTP within the cell. The cytoplasm is enriched with Ran:GDP, whereas Ran:GTP is more abundant in the nucleus [22]. Once cargo-IMP complexes reach the nucleoplasm, interaction between IMP*β*1 (or its orthologues) and Ran:GTP induces conformational changes that trigger cargo release. The IMPs are then recycled back to the cytoplasm to participate in additional rounds of nuclear import [23].

Kaposi’s sarcoma-associated herpesvirus (KSHV), a member of the *Gammaherpesvirinae* subfamily, establishes lifelong latent infections within host cells by maintaining its genome as an episomal molecule in the nucleus [24,25]. A pivotal factor in this process is the LANA, a multifunctional viral protein essential for episome maintenance, transcriptional regulation and immune evasion [26,30]. Upon nuclear entry, KSHV LANA binds to specific DNA sequences within the terminal repeats of the viral genome, a mechanism that is reminiscent of Epstein–Barr virus nuclear antigen 1 (EBNA1)’s role in the maintenance of the Epstein–Barr virus (EBV) genome [28,29]. This interaction enables LANA to tether the viral episome to host chromatin, ensuring its stable retention and equal segregation during host cell division – a process fundamental to the maintenance of viral latency [28,30]. In addition, LANA regulates the transcription of both host and viral genes [31,32]. Specifically, LANA binds to the tumour suppressor protein p53 and suppresses its transcriptional activity [33]. Additionally, LANA upregulates and activates the pro-survival protein survivin, which not only promotes cell survival but also influences viral replication by inhibiting histone deacetylase [34]. To perform all of these functions, LANA needs to enter the nucleus. One of the critical features enabling LANA’s nuclear localization is its cNLS, which allows recognition by the host cell’s IMP*α*/*β*1 transport machinery [35,37]. This interaction facilitates the active transport of LANA through the NPC into the nucleus, where it performs its essential functions. Alternatively, an atypical Arg/Gly-rich NLS located at the N-terminus of LANA has also been characterized, which directly interacts with IMP*β*1 to facilitate a non-classical nuclear import pathway [35].

The OvGHV-2 genome encodes a putative LANA orthologue, designated ORF73, predicted to function similarly to KSHV and AlHV-1 LANA based on sequence homology [9,38]. However, the functional characterization of OvGHV-2 ORF73, particularly its role in nuclear localization, episome tethering and potential involvement in viral genome nuclear import, remains entirely unexplored. This knowledge gap is primarily due to the inability of OvGHV-2 to replicate in established cell culture systems, which has significantly limited the study of its molecular biology and pathogenesis. In this study, we aimed to characterize the structural and functional properties of the predicted NLS within the LANA homologue of OvGHV2 and to elucidate the mechanisms governing its nuclear import. To achieve this, we employed an integrated approach combining structural biology techniques with quantitative biochemical assays and quantitative confocal laser scanning microscopy (CLSM) to explore the molecular interactions involved in OvGHV2 LANA homologue nuclear trafficking.

## Methods

### LANA homologue amino acid sequence comparison and phylogenetic analysis of *Gammaherpesvirinae*

The selected amino acid sequences of the LANA homologue of *Gammaherpesvirinae* available in GenBank were downloaded and stored in Geneious Prime (version 2025.1.1). Amino acid sequence similarity was calculated by MAFFT software (version 11.0.11) [39]. Amino acid sequences were aligned using MAFFT (version 7.450) with the G-INS-i algorithm (gap open penalty 1.53; offset value 0.123). Phylogenetic relationships within the subfamily *Gammaherpesvirinae* were inferred using predicted amino acid sequences obtained from the International Committee on Taxonomy of Viruses (ICTV) database. Sequence alignments were performed using muscle. Amino acid sequences corresponding to six conserved viral genes – uracil–DNA glycosylase, helicase–primase helicase subunit, DNA packaging terminase subunit 1, major capsid protein, envelope glycoprotein B and DNA polymerase catalytic subunit – were concatenated prior to analysis. Neighbour-joining phylogenetic trees were constructed from the multiple sequence alignments using the general time-reversible substitution model with 500 bootstrap replicates in Geneious Prime (version 2025.1.1). Viruses were labelled using their systematic virus names.

### Retrieval and analysis of OvGHV2 genomic sequences

Complete genome sequences of OvGHV2 were obtained from the GenBank database. Sequence analysis was conducted using Geneious Prime (version 2023.1.1). One ORF (designated ORF73 in National Center for Biotechnology Information (NCBI) Reference Sequence: NC_007646) was tentatively identified as a homologue of the latency-associated nuclear antigen (LANAh) [38]. To compare homologous sequences, multiple sequence alignment of the predicted ORF73 LANAh was performed using MAFFT (version 7.450), applying the G-INS-i strategy with a gap opening penalty of 1.53 and an offset value of 0.123.

### Peptide and gene construct design and synthesis

The ORF73 LANAh reference sequence from NCBI (YP_438196.1) was used to predict potential NLSs using the cNLS Mapper algorithm [39]. This analysis identified a bipartite NLS (^375^RGRRKRPPKHQPETDRAKRKKLAPIW^400^) with a prediction score of 8.7, indicating strong nuclear import potential. Synthetic peptides corresponding to the predicted NLSs, each modified with an FITC/Ahx group at the N-terminus, were synthesized at Macquarie University, Sydney, Australia, using standard Fmoc-based solid-phase peptide synthesis protocols on a CEM Liberty Blue^™^ automated synthesizer (CEM, USA) according to a previously published procedure [40]. Briefly, Rink amide resin was pre-swelled in a 1 : 1 mixture of dimethylformamide (DMF) and dichloromethane (DCM) for 1 h prior to synthesis. Amino acids were dissolved in DMF at a final concentration of 0.2 M and coupled sequentially from the C- to N-terminus at 90 °C for 3 min using five equivalents of amino acid, ten equivalents of N,N′-diisopropylcarbodiimide in DMF as activator and five equivalents of Oxyma/DIPEA (0.5 M/0.05 M in DMF) as base. Fmoc deprotection was performed with 20% piperidine in DMF at 90 °C for 2 min, followed by resin washing in DMF. Double couplings were employed for arginine residues to ensure complete incorporation. After final Fmoc removal of the N-terminal aminohexanoic acid (Ahx), the resin was washed and subjected to overnight coupling with three equivalents of fluorescein isothiocyanate (FITC) and six equivalents of DIPEA in DMF. Following FITC coupling, the peptides were sequentially washed with DMF, DCM and methanol before cleavage from the resin using a cocktail containing 92.5% trifluoroacetic acid, 2.5% triisopropylsilane, 2.5% thioanisole and 2.5% water for 3–6 h at room temperature. Peptides were then precipitated in ice-cold diethyl ether, dissolved in water, freeze-dried and purified using a Shimadzu LC-20AD high-performance liquid chromatography system (Shimadzu, Japan). Peptide identity and purity were confirmed by mass spectrometry on a Shimadzu LCMS-8050 instrument operating in positive electrospray ionization mode with a Polaris 3 C18-A 150×4.6 mm column (Agilent Technologies, USA) (Figs S4 and S5, available in the online Supplementary Material). Recombinant N-terminally truncated isoforms of importin *α*1 (hIMP*α*1ΔIBB), *α*2 (mIMP*α*1ΔIBB) and *α*3 (hIMP*α*3ΔIBB), lacking the autoinhibitory importin-*β* binding (IBB) domain, each incorporating a His-tag and TEV protease cleavage site, along with importin *β*1 encoded in the pMCSG21 vector, were produced as described below [41,42].

### Recombinant expression and purification of importin isoforms

Recombinant overexpression of importin isoforms *α*1, *α*2, *α*3, *α*7, *β*1, *β*2 and *β*3 was performed in *Escherichia coli* pLysS cells using an auto-induction protocol [43]. Cultures were incubated at room temperature for 36 h, and cells were harvested by centrifugation at 5,232 ***g*** for 30 min. Pellets were resuspended in His buffer A (50 mM phosphate, 300 mM NaCl, 20 mM imidazole, pH 8.0) and subjected to three freeze-thaw cycles, followed by enzymatic lysis with lysozyme (20 mg ml^−1^) and DNase (50 mg ml^−1^) at room temperature for 1 h. Clarified lysates were obtained by centrifugation (11,269 ***g***, 45 min) and filtration (0.45 µm membrane). Proteins were purified using HisTrap HP affinity chromatography with a linear imidazole gradient (20–500 mM), followed by size-exclusion chromatography on a HiLoad 26/60 Superdex 200 column equilibrated with GST buffer A (50 mM Tris, 125 mM NaCl, pH 8.0). Fractions containing the target proteins were pooled, concentrated (10 kDa MWCO filters), aliquoted and stored at –80 °C. Protein purity and integrity were assessed by SDS-PAGE (4–12% Bis-Tris Plus gel, 165 V, 35 min) and Coomassie staining.

### Crystallization, data collection and structure determination

Crystallization of IMPα2 was carried out using the hanging drop vapour diffusion method at 23 °C, following the protocol described previously [44]. Briefly, equal volumes of protein solution and reservoir solution (0.6 M sodium citrate, 0.1 M HEPES, pH 7.0 and 10 mM DTT) were mixed and equilibrated against 300 µl of reservoir solution. Rod-shaped crystals typically appeared within 48 h of incubation. Crystals were soaked with the desired peptide and cryoprotected in the reservoir solution containing 20% glycerol, before being flash frozen in liquid nitrogen. X-ray diffraction data were obtained from the Australian Synchrotron on the MX2 macromolecular beam lines [45] using the Eiger 16 M detector. Data were indexed and integrated using MOSFLM [46]. Merging, space-group assignment, scaling and R_free_ assignment were performed using AIMLESS within CCP4 [47,48]. Phasing was performed using molecular replacement in Phaser [49], and PDB code 1IQ1 was used as the search model for IMP*α*2. Final model building and refinement were performed using iterative cycles of COOT [50] and Phenix [51]. The finalized model was validated and deposited with the PDB (PDB ID: 9YTZ).

### Fluorescence polarization assay

Synthetic FITC-labelled peptide (2 nM final concentration) was incubated with twofold serial dilutions of importin isoforms, starting at 20 µM, across 23 wells in a final volume of 200 µl per well in GST buffer A (50 mM Tris, 125 mM NaCl, pH 8.0). Fluorescence polarization measurements were performed using a CLARIOstar Plus plate reader (BMG Labtech, Germany) in 96-well black Fluotrac microplates (Greiner Bio-One, Austria), essentially as previously [52,57]. Each assay was conducted in triplicate, each containing a negative control (no importin binding partner). Data were analysed with GraphPad Prism (Prism 9, Version 9.3.1), and a binding curve was fitted to the one-site-specific binding least squares fit function (accounts for ligand depletion) to determine the dissociation constant (Kd) and maximum binding (Bmax). Bmax data were not provided in the Fluorescence Polarization (FP) graph.

### Electro-mobility shift assay

A FITC-labelled peptide (10 µM) was incubated with 20 µM of each importin *α* isoform in a 20 µl reaction containing 3 µl of 50% (v/v) glycerol, with GST buffer A (50 mM Tris, 125 mM NaCl, pH 8.0) making up the remaining volume as previously described [57,58]. Samples were resolved on a 1% (w/v) agarose gel in TB buffer (45 mM Tris, 45 mM boric acid, pH ~8.5) at 80 V for 2 h. Gels were imaged using a SYBR Green filter on a Bio-Rad Gel Doc system to visualize FITC-peptide complexes. Subsequently, gels were stained with Coomassie Brilliant Blue R-250, destained overnight (10% ethanol, 10% acetic acid) and re-imaged to assess total protein migration and complex formation.

### Cell culture and transfections

HEK 293 A cells were cultured in Dulbecco’s modified Eagle’s medium (DMEM) with 10% (vol/vol) FBS, 50 U ml^−1^ penicillin, 50 U ml^−1^ streptomycin and 2 mM l-glutamine. The cells were kept in a humidified incubator at 37 °C with 5% CO_2_ and were passaged upon reaching confluence.

### CLSM and image analysis

HEK 293 A cells were seeded onto glass coverslips in a 24-well plate (5×10^4^ cells per well). The following day, the cells were transfected with varying amounts of expression constructs (100–250 ng) using Lipofectamine 2000 (Thermo Fisher Scientific, Monza, Italy), according to the manufacturer’s instructions. The cells were then incubated at 37 °C with 5% CO_2_ in complete medium [59] until they were ready for processing for CLSM. Twenty-four hours post-transfection, the cells were incubated for 30 min with DRAQ5 (#62251, Thermo Fisher Scientific; 1 : 5000 in DMEM without phenol red), washed twice with PHEM 1×buffer (60 mM PIPES, 25 mM HEPES, 10 mM EGTA and 4 mM MgSO_4_) and fixed with 4% (v/v) paraformaldehyde in PHEM for 10 min at room temperature. Following three washes with 1× PBS, coverslips were mounted on glass slides using Fluoromount G (Southern Biotech, Birmingham, AL, USA). The subcellular localization of GFP fusion proteins was analysed using a Nikon A1 confocal laser scanning microscope (Nikon, Tokyo, Japan) equipped with a 60× oil immersion objective, as previously described [60]. Levels of nuclear accumulation of proteins of interest were determined using the Fiji software (ImageJ) [61] from single-cell measurements of nuclear (Fn), cytoplasmic (Fc) and autofluorescence/background (Fb) fluorescence, following the subtraction using the formula Fn/c = (Fn-Fb) / (Fc−Fb), as previously described [52]. Graphs were prepared as described in [62] to allow visualization of both cell-to-cell and sample-to-sample variations. Statistical analysis of the data was performed using the Welch and Brown–Forsythe ANOVA test with GraphPad Prism 9 software.

## Results

### Genetic variability of the LANA homologue and phylogenetic relationships within the subfamily *Gammaherpesvirinae*

LANA is a multifunctional viral protein essential for episome maintenance, transcriptional regulation and immune evasion [28,30]. Upon nuclear entry, LANA tethers the viral episome to host chromatin, ensuring its stable retention and equal segregation during host cell division – a process fundamental to the maintenance of viral latency [29,30]. Despite its essential roles, the mechanisms underlying the nuclear trafficking of LANA remain poorly understood. To investigate this, we analysed representative gammaherpesvirus genomes and extracted full-length LANA homologue sequences for comparative analysis and further characterization.

**Fig. 1.**
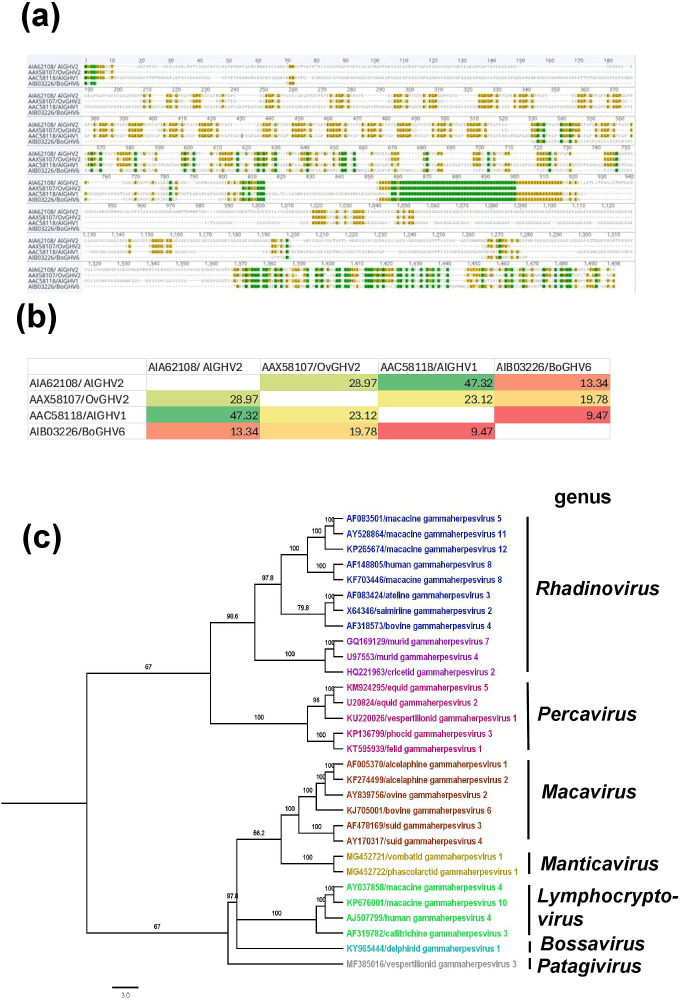
Multiple sequence alignment of the LANAh protein of selected *Macavirus* and evolutionary comparison of the members of the *Gammaherpesvirinae* subfamily. (**a**) LANA sequence alignment of four representative species of the *Macavirus* genus was performed in Geneious Prime (version 2025.1.1) using MAFFT (version 7.450) employing the G-INS-i algorithm, with a gap opening penalty of 1.53 and an offset value of 0.123. (**b**) Simplified image displaying the sequence identity of four representative virus species of the *Macavirus* genus. (**c**) Phylogenetic relationships within the subfamily *Gammaherpesvirinae*. Alignment of predicted amino acid sequences was adopted from ICTV, which was performed using muscle. Concatenation of the amino acid sequences predicted for six conserved genes (uracil–DNA glycosylase, helicase–primase helicase subunit, DNA packaging terminase subunit 1, major capsid protein, envelope glycoprotein B and DNA polymerase catalytic subunit) was used for the alignment. Neighbour-joining trees were constructed from multiple alignments of the predicted amino acid sequences using the general time-reversible substitution model with 500 bootstrap replicates in Geneious Prime (version 2023.1.1). The viruses are represented by systematic virus names.

The amino acid sequences of the LANA homologues from selected gammaherpesviruses exhibited pairwise identities ranging from 3.73 to 100% (Table S1). OvGHV-2 showed the highest identity with alcelaphine gammaherpesvirus 2 (27.75%), followed by alcelaphine gammaherpesvirus 1 (23.14%). Given the high divergence within members of the *Gammaherpesvirinae* subfamily, a separate alignment of LANA homologues from four representative viruses of the *Macavirus* genus – alcelaphine herpesvirus 1, alcelaphine herpesvirus 2, OvGHV2 and bovine gammaherpesvirus 6 – was performed. However, amino acid sequence identity remained highly variable (9.47–47.32%), and the predicted NLS sequence was not conserved across the alignment (Fig. 1a and b). Predicted NLS sequence of four representative viruses of the *Macavirus* genus is provided in the supplementary file (Fig. S1). These data indicate that neither the NLS sequence nor the NLS position is strongly conserved even within macaviruses. In addition, to place LANA nuclear import in a broader gammaherpesvirus context, we performed *in silico* NLS prediction for LANA homologues from diverse species to distinguish monopartite and bipartite NLS motifs. The protein length, predicted NLS types and NLS positions are summarized in Table S2. These analyses suggest that the observed NLS patterns vary independently among viruses rather than being strictly conserved. To further place this in an evolutionary context, our phylogenetic analysis (based on concatenated conserved herpesvirus genes rather than LANA alone) demonstrated that members of the *Macavirus* genus form a well-supported but highly divergent clade relative to other gammaherpesvirus genera (Fig. 1c). Macaviruses were clearly separated from *Rhadinovirus*, *Percavirus* and *Lymphocryptovirus* lineages, with longer branch lengths indicating substantial evolutionary divergence. This pronounced genetic separation suggests that macaviruses have undergone distinct evolutionary pressures, potentially leading to unique molecular mechanisms during infection. The marked divergence of macaviruses from other gammaherpesviruses highlights the likelihood of genus-specific host–virus interactions, supporting the necessity to investigate *Macavirus*-specific nuclear import pathways rather than extrapolating mechanisms from other gammaherpesvirus genera.

**Fig. 2.**
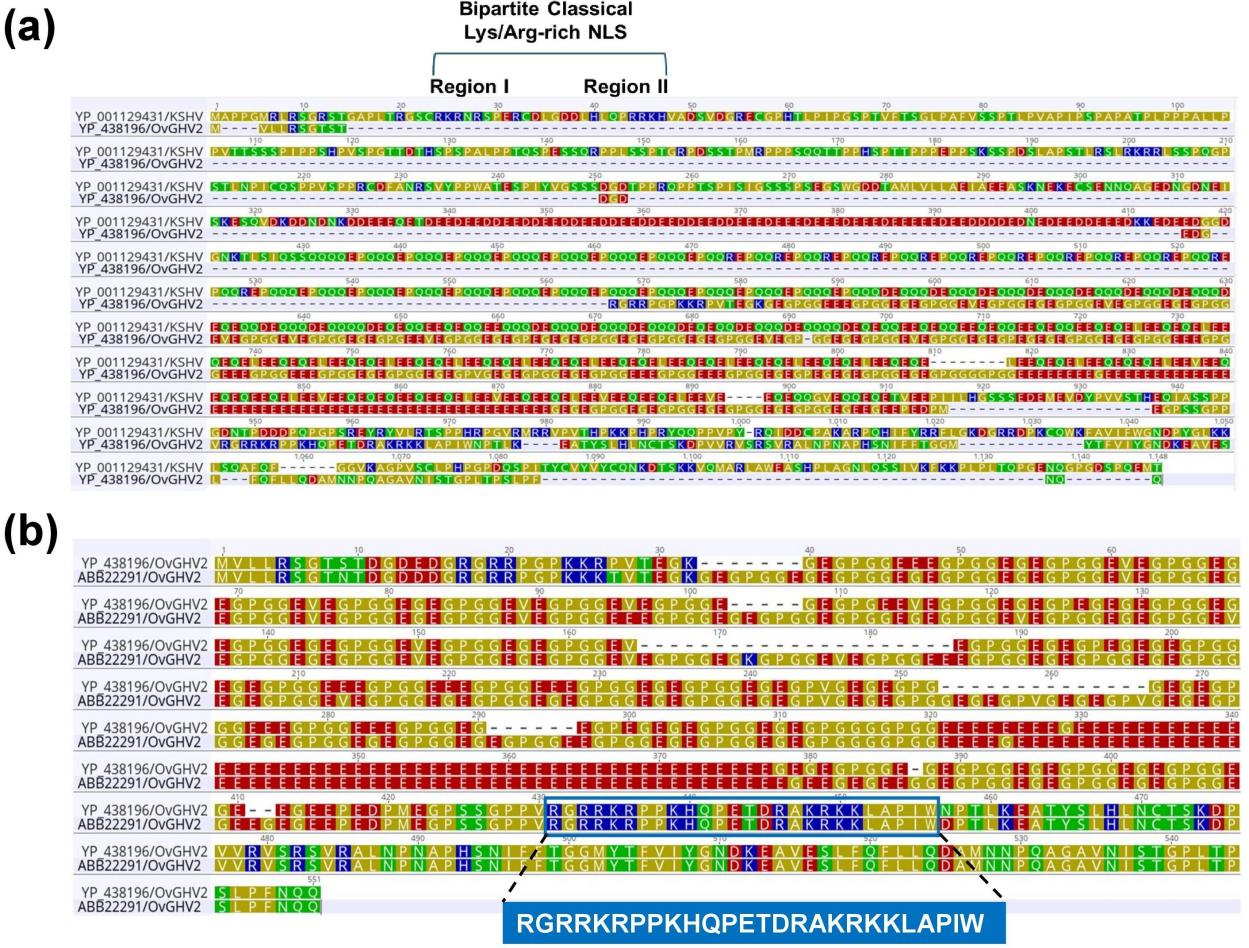
Amino acid sequence alignment of LANA homologues of the KSHV and OvGHV2. (**a**) Alignment of the full-length LANA homologues of the KSHV and OvGHV2 showing no obvious homology (11.67% amino acid sequence identity). The previously characterized bipartite lysine/arginine-rich NLS of KSHV [35] is indicated. The N-terminal part of KSHV LANA is rich in glutamate and aspartate residues, followed by a glutamine and aspartate-rich region in the central part, which is essential for long-term virus persistence. (**b**) The amino acid sequences of ORF73 LANA homologues from two OvGHV2 (ABB22291 and YP_438196) were aligned (85.12% sequence identity) to identify conserved motifs. The N-terminal part of OvHGV2 LANA is rich in glycine and proline, followed by a glutamate-rich region in the central part, an important property of Gammaherpesvirus genome maintenance proteins for enabling virus persistence [10]. The predicted conserved NLS identified by cNLS mapper with a score of 8.7 has been shown in the blue box. Sequence alignment was performed in Geneious Prime (version 2025.1.1) using MAFFT (version 7.450) employing the G-INS-i algorithm, with a gap opening penalty of 1.53 and an offset value of 0.123. The colour of the alignment was chosen based on polarity, where red indicates acidic residues and blue indicates basic residues.

A bipartite NLS was functionally characterized previously in KSHV/HHV-8 [35]. Therefore, amino acid sequence alignments were performed for KSHV and OvGHV2 LANA homologues to investigate conserved structural and functional motifs within LANA. Alignment of KSHV and OvGHV2 LANA homologues revealed no obvious sequence conservation (Fig. 2a). The alignment demonstrated the bipartite lysine/arginine-rich NLS in the N-terminal, previously characterized in KSHV LANA [35]. However, a putative cNLS (^375^RGRRKRPPKHQPETDRAKRKKLAPIW^400^) in the C-terminal region of OvGHV-2 LANA was predicted by cNLS mapper with a score of 8.7, indicating strong potential for nuclear targeting (Fig. 2b). The predicted cNLS score of 8.7 falls within the high-confidence range for classical nuclear localization signals in this prediction framework.

### Biochemical determination of OvGHV2 LANAh NLS preference for both human IMP*α* and IMP*β* isoforms

**Fig. 3.**
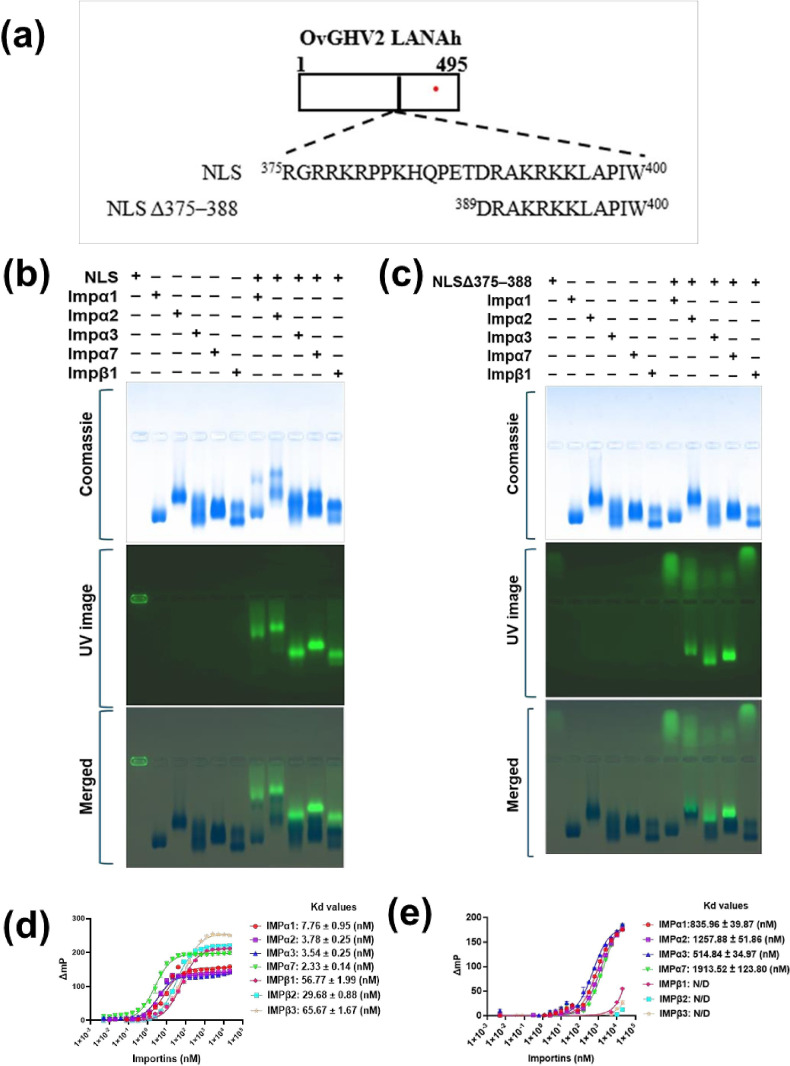
OvGHV2 LANAh NLS binds to several IMPs. (**a**) Schematic representation of the OvGHV2 LANAh NLS, including NLSΔ375–388, in which the upstream basic stretch of amino acids was deleted from the NLS. (**b**) EMSA showing binding of OvGHV2 LANAh NLS to IMP*α* isoforms. (**c**) EMSA showing binding of NLSΔ375–388 to IMP*α* isoforms. NLS peptides contain a FITC and Ahx linker and were visualized by excitation with a UV lamp (green). Proteins were stained using Coomassie blue stain. EMSA results are representative of three independent experiments. (**d**) FP assay measuring the binding affinity between the OvGHV2 LANAh NLS and respective IMP isoforms. (**e**) FP assay measuring the binding affinity between the NLSΔ375–388 and respective IMP isoforms. Data shown are mean±standard error of the mean (se) relative to three independent experiments. Dissociation constants (Kd values) were determined by conducting a saturation binding experiment using FITC-labelled peptide and importin proteins. The resulting data are then fitted to a binding curve using non-linear regression analysis in GraphPad Prism.

To evaluate whether the predicted NLS binds to host nuclear import receptors IMP*α* and IMP*β*, and to identify any preference for specific receptor isoforms, we conducted biochemical binding assays. Electro-mobility shift assays (EMSAs) were performed to qualitatively assess the interactions between the OvGHV2 LANAh NLS and various importin isoforms, including IMPα family members (*α*1, *α*2, *α*3 and *α*7) and IMP*β*1. Three independent experiments confirmed that the predicted NLS was capable of binding to IMP*α* isoforms as well as to IMP*β*1, as indicated by the shift of the FITC-labelled NLS peptide after binding to IMPs (Fig. 3b). On the other hand, binding of the OvGHV2 LANAh NLS is reduced after deletion of the upstream portion of the NLS (NLSΔ375–388), which contains the basic residues ^377^RRKR^380^ (Fig. 3c).

To further assess the interactions and determine the binding affinities of the IMP/peptide complexes, quantitative FP assays were performed. The NLS demonstrated higher binding affinity to IMP*α* isoforms compared to IMP*β*s. The highest affinity was measured for IMP*α*7 (Kd=2.33 nM), followed by IMP*α*3 (Kd=3.54 nM), IMP*α*2 (Kd=3.78 nM), IMP*α*1 (Kd=7.76 nM), IMP*β*2 (Kd=29.68 nM), IMP*β*1 (Kd=56.77 nM) and IMP*β*3 (Kd=65.67 nM) (Fig. 3d). In contrast, the NLSΔ375–388 demonstrated much lower binding affinities compared to the full-length OvGHV2 LANAh NLS, interacting with them accordingly: IMP*α*3 Kd=514.84 nM, IMP*α*1 Kd=835.96 nM and IMP*α*2 Kd=1257.88 nM, followed by IMP*α*7 Kd=1913.52 nM (Fig. 3e). Interestingly, no detectable binding affinity was observed with all IMP*β* isoforms after removal of the upstream basic stretch of amino acids. These findings indicate that both N- and C-terminal NLS motifs are important to stabilize the interaction with IMPα and support the hypothesis that OvGHV2 LANAh can bind to either the IMP*α* adapter or directly to the IMP*β* nuclear import receptors to mediate nuclear import.

### OvGHV2 LANAh NLS binds to IMP*α* with a bipartite NLS

**Fig. 4.**
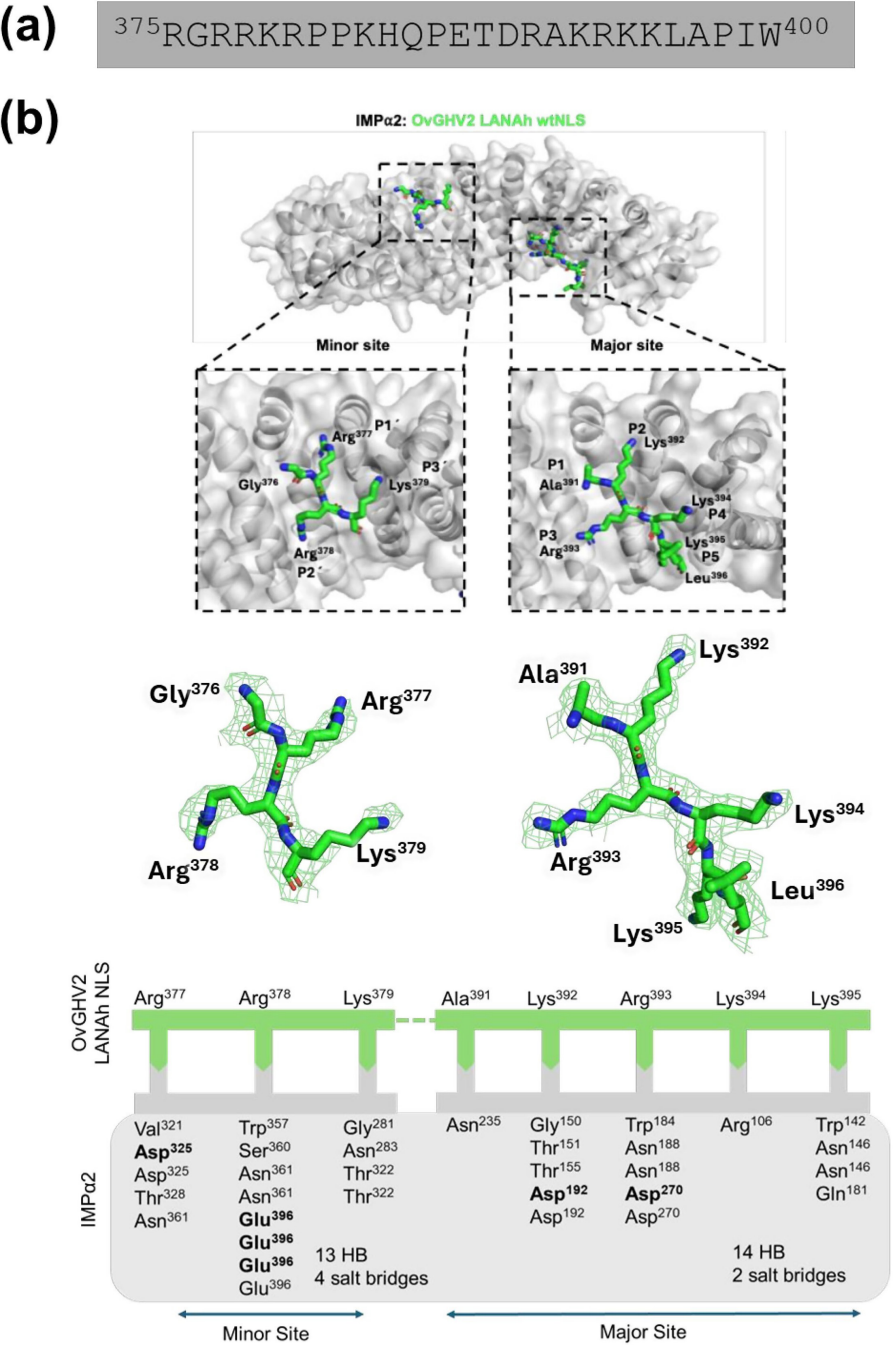
Crystal structure and binding interactions of OvGHV2 LANAh NLS in complex with mouse IMP*α*2. (**a**) Sequence of the predicted NLS of OvGHV2 LANAh. (**b**) Top panel: structure of OvGHV2 LANAh NLS (green sticks) and IMP*α*2 (grey surface) complex resolved to 2.4 Å resolution. The zoomed-in images illustrate critical residues of OvGHV2 LANAh NLS binding in both minor and major IMP*α*2 sites. Middle panel: electron density maps showing the peptide fitted into the density at the binding site. This structure has been deposited in the PDB and given the code: 9YTZ. Bottom panel: simplified representation of IMP*α*2 and OvGHV2 LANAh NLS binding interactions. The OvGHV2 NLS (green line) residues bound to IMP*α*2 (grey box) are indicated through complementary arrows. Residues in bold denote salt bridges, and non-bold residues indicate hydrogen bonds identified using the PDBePISA server.

We undertook X-ray crystallography to gain an understanding of the interactions between OvGHV2 LANAh protein and the nuclear transport receptor IMP*α* by crystallizing IMP*α*2 with FITC-tagged NLS peptide (^375^RGRRKRPPKHQPETDRAKRKKLAPIW^400^). The diffraction data were indexed to the space group *P*2_1_ 2_1_ 2_1_ with the unit cell parameters 78.5 90.1 100.4 (Table 1). The structure was resolved to 2.4 Å, and the resulting model has a single molecule of IMP*α* and the OvGHV2 LANAh NLS (detailed statistics can be seen under Table 1). Structural analysis revealed that OvGHV2 LANAh NLS acts as a bipartite NLS, which binds to IMP*α*2 in both the major and minor sites (Fig. 4). The IMP*α*2 minor binding site accommodates the OvGHV2 LANAh protein NLS sequence ^377^RRK^379^, with Arg^378^ occupying the P2 site (comprised of Glu^396^, Trp^357^, Ser^360^ and Asn^361^). The interaction is mediated by 13 hydrogen bonds and 4 salt bridges. On the other hand, the IMP*α*2 major binding site accommodates the OvGHV2 LANAh protein NLS sequence ^391^AKRKK^395^, with Lys^392^ occupying the P2 site (comprised of Asp^192^, Gly^150^, Thr^151^ and Thr155). The interaction is mediated by 14 hydrogen bonds and 2 salt bridges (Table 2), and interacting residues are strongly conserved in ovine IMP*⍺*1 (Fig. S3).

**Table 1.** Data collection and refinement statistics for the structure of mouse IMP*α*2 in complex with OvGHV2 LANAh NLS

OvGHV2 LANAh NLS and mouse IMP*α*2 (PDB code: 9YTZ)	
Data collection (high-resolution statistics in parentheses)	
Wavelength	0.95366
Data collection temperature (K)	100
Detector type	DECTRIS EIGER X 16M
Detector	PIXEL
Resolution range (Å)	29.6–2.85 (3.0–2.85)
Space group	*P* 2_1_ 2_1_ 2_1_
Unit cell (Å)	78.5 90.1 100.4
Total reflections	129185 (19287)
Unique reflections	17239 (2485)
Multiplicity	7.5 (7.8)
Completeness (%)	99.9 (100)
Mean I/σ (I)	4.9 (1.4)
Wilson B-factor Å^2^	31.7
R_pim_	0.211 (1.155)
CC(1/2)	0.960 (0.446)
**Refinement**	
Reflections used in refinement	17195 (1689)
Reflections used for R-free	870 (89)
R_work_	0.2280 (0.3293)
R_free_	0.2647 (0.3782)
No. of non-hydrogen atoms	3330
Solvent	0
Protein residues	436
Bond length r.m.s.d (Å)	0.002
Bond angle r.m.s.d (^o^)	0.48
Ramachandran favoured (%)	98.14
Ramachandran allowed (%)	1.86
Ramachandran outliers (%)	0.00
Average B-factor	42.94

**Table 2.** Hydrogen bond and salt bridge interactions between OvGHV2 LANAh NLS and mouse IMP*α*2

Mouse IMP*α*2	OvGHV2 LANAh NLS
**Hydrogen bonds (minor site**)	
VAL 321[O]	ARG 377[NH1]
ASN 361[O]	ARG 377[NH1]
ASP 325[OD1]	ARG 377[NH2]
THR 328[OG1]	ARG 377[NH2]
ASN 361[OD1]	ARG 378[N ]
SER 360[OG]	ARG 378[NH1]
GLU 396[OE1]	ARG 378[NH2]
GLY 281[O]	LYS 379[NZ]
ASN 283[OD1]	LYS 379[NZ]
THR 322[O]	LYS 379[NZ]
THR 322[OG1]	LYS 379[ Z]
TRP 357[NE1]	ARG 378[O ]
ASN 361[ND2]	ARG 378[O]
**Salt bridges (minor site**)	
ASP 325[OD1]	ARG 377[NH2]
GLU 396[OE1]	ARG 378[NH1]
GLU 396[OE1]	ARG 378[NH2]
GLU 396[OE2]	ARG 378[NH2]
**Hydrogen bonds (major site**)	
ASP 192[OD1]	LYS 392[NZ]
GLY 150[O]	LYS 392[NZ]
THR 151[O]	LYS 392[NZ]
THR 155[OG1]	LYS 392[NZ]
ASN 188[OD1]	ARG 393[N ]
ASP 270[OD2]	ARG 393[NH2]
ARG 106[O ]	LYS 394[NZ]
ASN 146[OD1]	LYS 395[N]
GLN 181[OE1]	LYS 395[NZ]
ASN 235[ND2]	ALA 391[O]
ASN 188[ND2]	ARG 393[O]
TRP 184[NE1]	ARG 393[O]
TRP 142[NE1]	LYS 395[O]
ASN 146[ND2]	LYS 395[O]
**Salt bridges (major site**)	
ASP 192[OD1]	LYS 392[NZ]
ASP 270[OD2]	ARG 393[NH2]

### OvGHV2 LANAh NLS shows less affinity to both IMP*α*1 major and minor site mutants

**Fig. 5.**
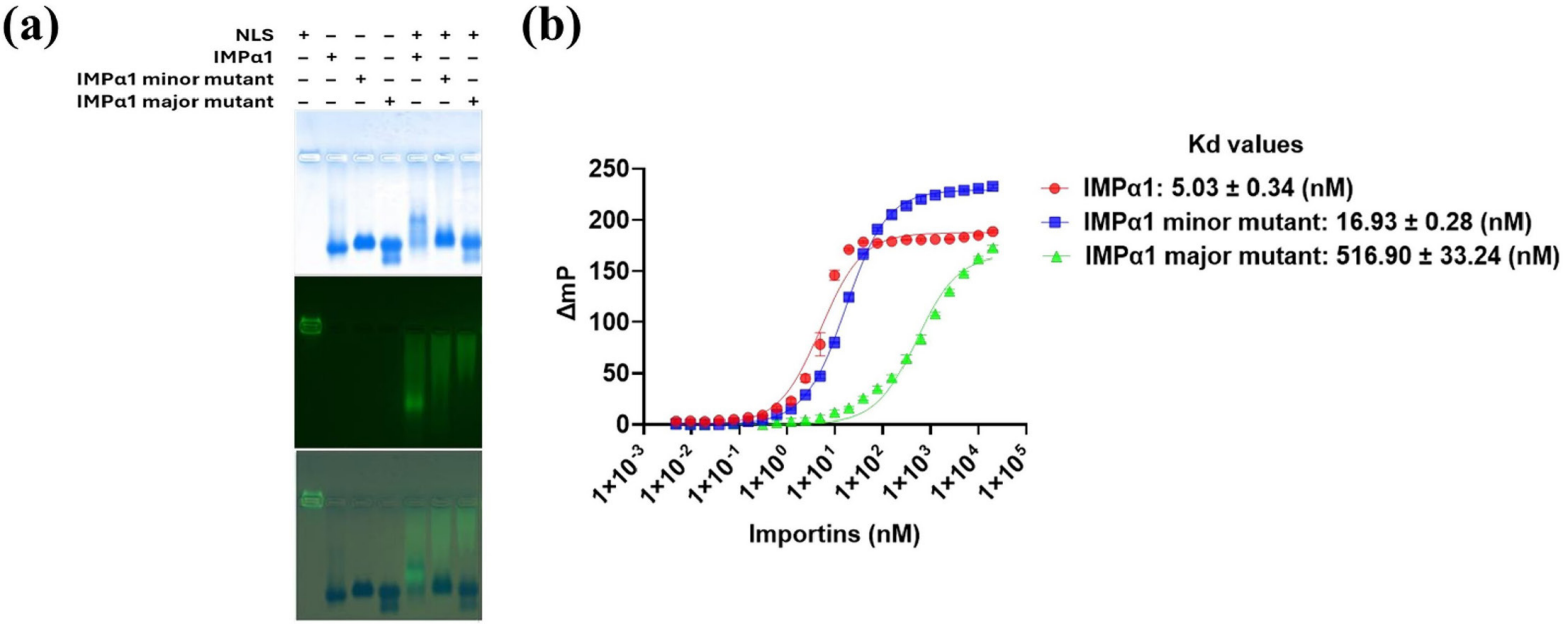
OvGHV2 LANAh NLS binds to IMP*α*1 mutants. (**a**) EMSA showing binding of the NLS to IMP*α*1 mutants. (**b**) FP assay measuring the binding affinity between the OvGHV2 LANAh NLS and IMP*α*1 mutants. Data shown are mean±standard error of the mean relative to three independent experiments. Data were used to calculate the Kd, as described in the ‘Methods’ section.

To confirm the binding mode of the OvGHV2 LANAh NLS, we investigated its interaction with IMPα1 mutants carrying point substitutions in either the major or minor binding sites using EMSA and FP assays (Fig. 5). Substitution of Asp^192^ with Lysine (D^192^K) in the major binding site of IMPα1 resulted in reduced co-migration with the OvGHV2 LANAh NLS in EMSA and less binding affinity, as shown by a threefold increase in the Kd value in FP analysis compared to wild-type IMP*α*1. Similarly, mutation of Glu^396^ to arginine (E^396^R) in the minor binding site also diminished co-migration in EMSA and led to a 100-fold increase in the Kd value relative to the wild-type IMP*α*1. These findings confirm that OvGHV2 LANAh NLS interacts with both the major and minor binding sites of IMPα1, contributing to the stabilization of the NLS-IMP complex.

### OvGHV2 LANAh is transported into the nucleus by the IMP*α*/*β*1 heterodimer

**Fig. 6.**
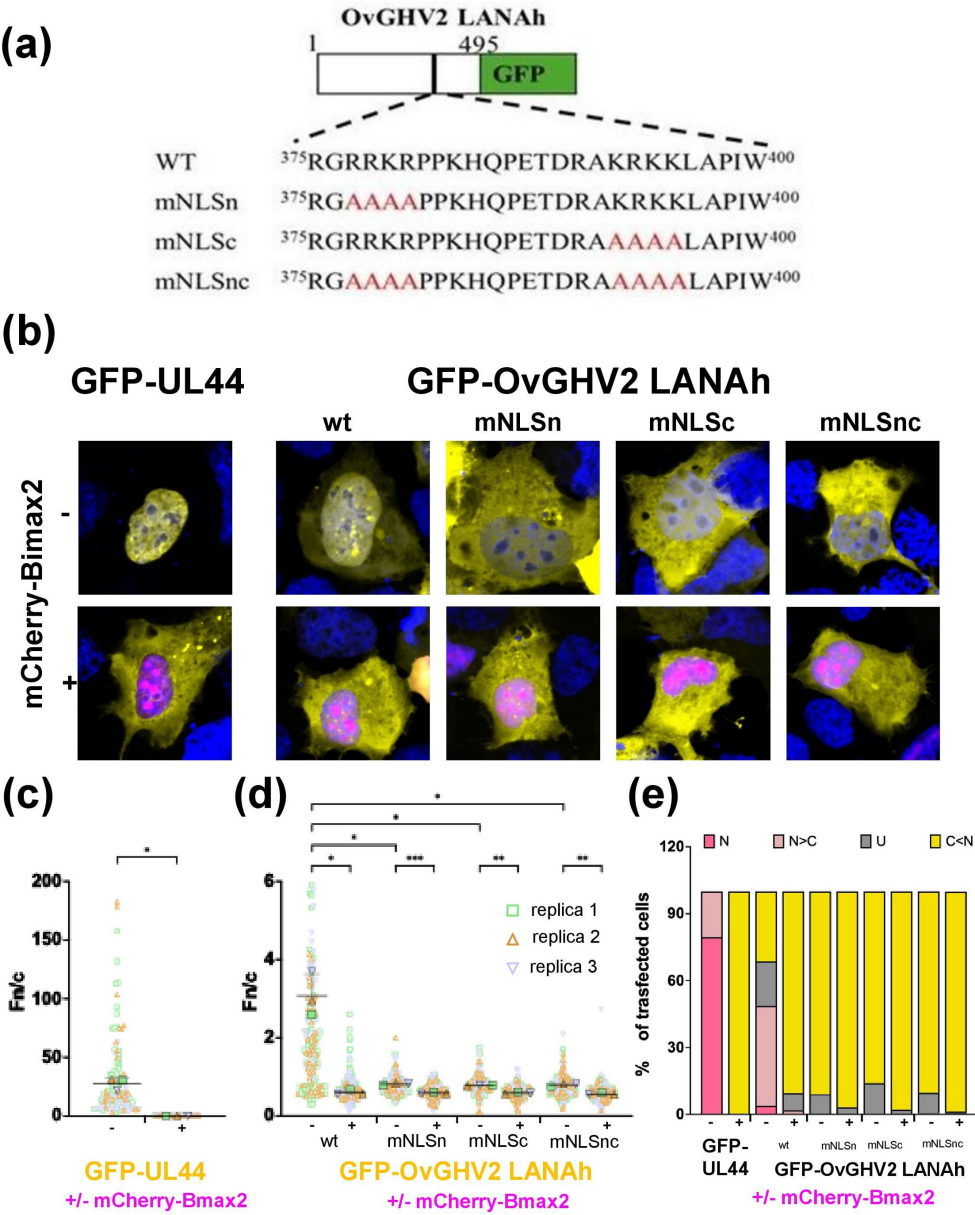
OvGHv2 LANAh nuclear localization is mediated by the IMP*α*/*β*1 heterodimer. (**a**) HEK 293 A cells were seeded on glass coverslips and transfected to express the indicated GFP fusion proteins in the presence or absence of a plasmid mediating the expression of mCherry-Bimax2. Sequences include mutated residues in red. (**b**) Twenty-four hours later, cells were treated with DRAQ5 to stain cell nuclei, fixed and mounted on microscope slides to allow quantitative CLSM analysis. Representative images of the indicated GFP fusion proteins expressed in the absence (top panels, ‘-’) or presence (bottom panels, ‘+’) of mCherry-Bimax2. Merged images of the GFP, mCherry and DRAQ5 channels are shown in yellow, magenta and blue, respectively. (**c, d**) Micrographs, such as those shown in panel b, from three independent experiments were quantitatively analysed to calculate Fn/c relative to each GFP fusion protein at the single-cell level. Individual measurements and means of each biological replica are shown as symbols coloured according to the biological replica, and the mean values of the three biological replicas are shown as horizontal lines, with sds indicated as vertical lines. Results from the Welch and Brown–Forsythe ANOVA test of significance are shown. -, Cells non-transfected with mCherry-Bimax2; +, cells transfected with mCherry-Bimax2; *P<0.05, **P<0.005, ***P<0.0005. (**e**) The percentage of cells relative to each indicated GFP fusion protein, displaying the indicated subcellular localization, is shown. N, nuclear, Fn/c ≥ 10; *N* > C, nuclear more than cytosolic, 2 ≤ Fn/c < 10; U, ubiquitous, 1 ≤ Fn/c < 2; C > N, more cytosolic than nuclear, Fn/c < 1.

To investigate the nuclear localization of the GFP-tagged full-length OvGHV2 LANAh protein and the role of host nuclear import factors, we conducted transfection assays in HEK 293 A cells in the presence or absence of the IMP*α*/*β*1 inhibitor Bimax2 (Fig. 6). Following transfection, confocal imaging revealed that the OvGHV2 LANAh protein localized both in the nucleus and in the cytoplasm, with weak nuclear accumulation, as indicated by a nuclear-to-cytoplasmic fluorescence ratio (Fn/c) of ~1.8, indicating active nuclear import. However, treatment with Bimax2 significantly reduced nuclear accumulation of LANAh (Fn/c ≈ 0.8), suggesting that IMP*α*/*β*1 plays a critical role in mediating its nuclear transport. Since our data suggest that OvGHV2 LANAh residues (^375^RGRRKRPPKHQPETDRAKRKKLAPIW^400^) represent a bipartite NLS, we assessed the impact of the substitution of basic residues within the upstream and downstream basic stretch of amino acids on its nuclear localization (mNLSn: ^375^RGAAAAPPKHQPETDRAKRKKLAPIW^400^, mNLSc: ^375^RGRRKRPPKHQPETDRAA

AAALAPIW^400^ and mNLSnc: ^375^RGAAAAPPKHQPETDRAAAAALAPIW^400^). Importantly, substitution of either stretch of basic amino acids was sufficient to strongly impair OvGHV2 LANAh nuclear import, causing an exclusion of the protein from the nucleus in almost 100% of transfected cells (Fig. 6c, d and an average Fn/c ≈ 0.8, significantly lower than that measured for the wild-type protein (Fig. 6e). Intriguingly, co-expression with mCherry-Bimax2 further decreased but did not completely abolish nuclear localization of the NLS mutant derivatives, and residual nuclear localization persisted (expected Fn/c ≈ 0.2 with cytoplasmic controls) (Fig. S2), indicating that nuclear import was not fully abolished. Given the molecular weight of the OvGHV2 LANAh protein (~75 kDa), passive diffusion is unlikely. Therefore, these data suggest the possible involvement of additional NLSs mediating an alternative IMP*α*/*β*1-independent nuclear import pathway that may partially compensate for disrupted classical nuclear import mechanisms. Collectively, these results demonstrate that the nuclear import of OvGHV2 LANAh is mediated predominantly by the recognition of a bipartite cNLS by the IMP*α*/*β*1 heterodimer. However, the partial nuclear presence under inhibitory or mutational conditions suggests that non-canonical transport mechanisms may also contribute to its nuclear transport.

## Discussion

We identified the ORF73 LANA homologue (LANAh) of OvGHV2, which infects hosts within the order Artiodactyla, including bison, cattle and cervids. Furthermore, a bipartite cNLS was predicted in the C-terminal region of the OvGHV2 LANAh using the cNLS Mapper (Fig. 1b). Our results demonstrate that this NLS binds multiple IMP*α* isoforms with high affinity and, to a lesser extent, IMPβs. The bipartite nature of the NLS was confirmed by biochemical (Fig. 3), structural (Fig. 4) and functional assays (Fig. 6), indicating that it simultaneously binds to IMP*α* with two basic stretches of amino acids, which are both required for efficient nuclear accumulation. Moreover, the observed nuclear localization in the transfection assays in the absence of the predicted NLS, together with its inhibition by Bimax2, suggests the presence of an alternative NLS elsewhere in OvGHV2 LANA that was not identified by the cNLS Mapper. This observation highlights the limitation of sequence-based NLS prediction tools and indicates that OvGHV2 LANA likely contains a non-canonical or structurally dependent NLS motif that mediates IMP*α*-dependent nuclear transport. Proteins can contain multiple or overlapping NLSs that contribute to their nuclear localization, highlighting the diversity of NLS motifs [63,64].

Our comparative sequence analyses place the OvGHV2 ORF73/LANA nuclear import findings into a broader evolutionary framework. LANA homologues across the *Gammaherpesvirinae* are highly divergent, with pairwise amino acid identities ranging from 3.73 to 100%, indicating strong lineage-specific variation (Table S1). Even within the genus *Macavirus*, representative LANA homologues show limited conservation (9.47–47.32% identity), and predicted NLS motifs are not positionally or compositionally conserved (Fig. 1a and b). Consistent with this, our concatenated-gene phylogeny demonstrates marked divergence of macaviruses from other *Gammaherpesvirus* genera (Fig. 1c), suggesting genus-specific host–virus interactions and supporting the need to investigate *Macavirus* nuclear import mechanisms directly rather than extrapolating from rhadinovirus or lymphocryptovirus models. In our current comparative analysis across the subfamily, it would be difficult to ascertain that predicted monopartite vs. bipartite NLS architecture and N- vs. C-terminal NLS positioning do not clearly partition with established phylogeny, although OvGHV2 contains a functional bipartite C-terminal NLS (Fig. S1 and Table S2). Because LANA sequences vary substantially in length and align poorly across genera, LANA-based phylogeny and NLS trait mapping might not be robust. These observations support a model in which LANA nuclear import strategies are evolutionarily flexible, with functional validation required to define NLS usage across macaviruses and other gammaherpesvirus lineages.

KSHV/HHV-8 LANA contains a previously functionally characterized bipartite NLS [35]; therefore, we performed amino acid sequence alignments between KSHV and OvGHV2 LANA (ORF73) proteins to examine conservation of structural and functional motifs. The alignment showed no obvious overall sequence conservation between the two homologues (Fig. 2a), although the known N-terminal lysine/arginine-rich bipartite NLS was evident in KSHV LANA. In contrast, no comparable N-terminal motif was observed in OvGHV2 LANA. Instead, cNLS Mapper predicted a strong classical NLS in the C-terminal region of OvGHV2 LANA (375-400; RGRRKRPPKHQPETDRAKRKKLAPIW) with a score of 8.7, which falls within the high-confidence range for nuclear targeting (Fig. 2b). High cNLS prediction scores in this range are typical of experimentally validated classical monopartite and bipartite NLS motifs, including benchmark signals, such as SV40 large T-antigen and other lysine/arginine-rich IMPα binding sequences [20,39, 65]. Notably, a structurally characterized C-terminal cNLS has also been reported for EBV EBNA1, supporting the possibility that gammaherpesvirus latency proteins may utilize distinct NLS architectures [66].

Our biochemical binding assays revealed that the OvGHV2 NLS binds all tested isoforms of IMP*α* (*α*1, *α*2, *α*3 and α7), as well as IMP*β*s, with varying affinities (Fig. 3). Among the IMPs, the NLS showed the highest affinity for IMPα7 (Kd=2.33 nM), followed by *α*3 (Kd=3.54) and *α*2 (Kd=3.78), while its interaction with IMPβs was comparatively weaker. For comparison with our FP data, previous work demonstrated that the black sea bass polyomavirus large tumour antigen (BSB PyV-LTA) NLS binds to all tested importins, with markedly higher affinity for IMP*α*7, IMP*α*5 and IMP*α*2 (Kd values were recorded in the low nanomolar range) compared with IMP*β*1–3, for which accurate Kd values could not be determined due to minimal binding. These findings indicate that BSB PyV-LTA residues 660–683 confer nuclear localization to a heterologous protein and preferentially engage IMP*α* isoforms, supporting their classification as a bona fide classical NLS [67]. In contrast, the human parvovirus B19 non-structural protein is also transported to the nucleus via importin *α*/*β*-dependent nuclear import, and FP analysis showed that the NS1 (172–182) peptide binds IMP*α*2ΔIBB with relatively low affinity (Kd ≈ 1 µM) [52]. While some viral proteins are known to show IMP*α* isoform preferences [68,70], our data indicate that the OvGHV2 ORF73 NLS displays broad isoform compatibility rather than strong selectivity. Also, direct interaction with IMP*β*1 in the FP assay supports the possibility of a non-classical nuclear import pathway, as reported for other viral proteins, including those from HIV-1 and influenza virus [71,72].

Structural analysis of the OvGHV2 LANAh NLS bound to mouse IMP*α*2 provided molecular insight into its bipartite nature (Fig. 4). The crystallographic data revealed dual engagement of the major and minor binding sites of IMP*α*2, mediated by distinct clusters of basic residues (^391^AKRKK^395^ and ^377^RRK^379^). These interactions were stabilized by numerous hydrogen bonds and salt bridges, consistent with high-affinity binding. Such bipartite recognition is characteristic of classical NLS motifs seen in cellular and viral proteins [73,74]. Mutational analysis of human IMP*α*1 further supported this model (Fig. 5). Substitution of Asp^192^ (major site) or Glu^396^ (minor site) significantly impaired binding in both EMSA and FP assays. Notably, the Asp^192^ mutation led to a dramatic 100-fold reduction in affinity, suggesting a critical contribution of the major site to overall binding stability. This observation is consistent with the behaviour of most classical bipartite NLSs, which predominantly depend on the major site [75], suggesting that OvGHV2 LANAh may employ a conventional NLS architecture to enhance importin engagement.

Transfection studies provided new insights into the nuclear import mechanisms of the OvGHV2 LANAh protein and highlighted the complexity of its intracellular trafficking. The strong reduction in nuclear accumulation upon IMP*α*/*β* inhibition by Bimax2 or NLS mutation confirms that classical nuclear import pathways are primarily responsible for OvGHV2 LANAh translocation into the nucleus (Fig. 6). However, the persistence of partial nuclear localization under the above-mentioned conditions suggests that there could be another NLS that mediated some interaction with other IMPs. In addition to classical nuclear localization signals, a substantial number of proteins are transported to the nucleus through atypical targeting motifs that do not conform to canonical Lys/Arg-rich consensus sequences [76]. These motifs are collectively described as ncNLS. One well-characterized subgroup is the proline-tyrosine type, referred to as PY-NLS. Previous work by Wang *et al.* identified a PY-NLS motif (residues 66–92) at the N-terminus of the human cytomegalovirus UL79 protein, showing strong similarity to the human PY-NLS consensus architecture, including a characteristic C-terminal PY core preceded by hydrophobic residues [77]. Comparable PY-NLS-like motifs have also been described in cellular proteins, such as the C-terminal region of the Ewing sarcoma protein [78], where they play a critical role in mediating nuclear import. Further studies are warranted to identify the additional host factors involved and to determine whether this alternative import contributes to viral persistence, latency or immune evasion *in vivo*. Interestingly, comparisons can be drawn between OvGHV2 LANAh and other gammaherpesvirus latency-associated nuclear antigens, particularly the KSHV LANA. Studies have shown that KSHV LANA localizes to the nucleus of latently infected cells and associates with chromatin during both interphase and mitosis [26,27]. An NLS was mapped to the N-terminal region of KSHV LANA, and this motif is similar to NLS sequences in EBV EBNA1, sharing regulatory phosphorylation sites for protein kinase A and cdc2-like kinases [36,79]. These phosphorylation events near or within the NLS have been shown to modulate nuclear import efficiency in other systems [80,81]. Additionally, the N-terminal region of KSHV LANA NLS can independently promote localization of reporter proteins to the nucleus, with the fusion protein forming discrete nuclear speckles, similar to the punctate pattern of full-length protein [33,82]. Whether OvGHV2 LANAh follows a similar localization pattern or undergoes cell cycle-regulated nuclear trafficking remains an intriguing question for future investigation.

A limitation of this study is that the binding assays could not be performed with full-length OvGHV2 LANAh protein, most likely due to its large size, the complexity of fluorescent labelling and associated technical challenges in recombinant expression. Instead, we used a fluorescently labelled synthetic peptide corresponding to the predicted NLS, which specifically interacted with nuclear import receptors, providing functional evidence for its role in nuclear import. Future studies using truncated or full-length constructs will be important to further define the mechanistic details of OvGHV2 LANAh–importin interactions. Another limitation of this study is that we used human and mouse IMP*α* isoforms rather than sheep-derived proteins. Although IMP*α* proteins are highly conserved between sheep and human/mouse (Fig. S3), particularly in the ARM repeat domain that mediates NLS binding, and cross-species compatibility of IMP*α*–NLS interactions has been widely demonstrated [53,57, 58], it would be important to investigate further using sheep IMP*α* to confirm host-specific interactions and strengthen the virological significance of our findings. While our structural, biochemical and confocal imaging data establish that the OvGHV2 LANA NLS mediates importin *α*/*β*1-dependent nuclear import, quantitative single-cell nuclear import kinetic measurements would provide additional insight into the dynamics, heterogeneity and potential alternative pathways of LANA nuclear entry. Such real-time kinetic analyses represent an important direction for future studies to further refine the mechanistic understanding of OvGHV2 LANA nuclear trafficking.

In summary, this study demonstrates that the predicted NLS within the OvGHV2 LANAh facilitates nuclear import via the IMP*α*/*β*1-dependent pathway, while also suggesting the potential involvement of alternative NLS in the OvGHV2 LANAh, which might contribute to nuclear localization. The results indicate that herpesviruses may utilize multiple, possibly species-specific, nuclear transport routes to achieve efficient nuclear targeting. To further elucidate the mechanisms underlying this process, future studies should focus on detailed subcellular localization analyses and the exploration of additional nuclear import pathways. Moreover, although this investigation centred on the OvGHV2 LANAh, it would be valuable to assess whether comparable nuclear import strategies are conserved across other gammaherpesviruses by examining their respective LANAh. Collectively, these findings enhance our understanding of OvGHV2 molecular biology and provide insights that could inform the development of targeted antiviral strategies and rational vaccine design, while contributing to the broader field of animal herpesvirus research.

## Supplementary material

10.1099/jgv.0.002250 Fig. S1.

## Abbreviations

AlHV-1: alcelaphine herpesvirus 1
CLSM: confocal laser scanning microscopy
cNLS: classical nuclear localization signal
DCM: dichloromethane
DMEM: Dulbecco’s modified Eagle’s medium
DMF: dimethylformamide
EBNA1: Epstein–Barr virus nuclear antigen 1
EBV: Epstein–Barr virus
EMSAs: electro-mobility shift assays
Fb: autofluorescence/background
Fc: cytoplasmic
FITC: fluorescein isothiocyanate
Fn: nuclear
IBB: importin-*β* binding
IMP: importin
IMP*α*: importin alpha
KSHV: Kaposi’s sarcoma-associated herpesvirus
LANA: latency-associated nuclear antigen
LANAh: homologue of the latency-associated nuclear antigen
MCF: malignant catarrhal fever
ncNLSs: non-classical nuclear localization signals
NLS: nuclear localization signal
NLSs: nuclear localization signals
NPC: nuclear pore complex
OvGHV2: ovine gammaherpesviruses 2

## References

[1] Li H, Cunha CW, Davies CJ, Gailbreath KL, Knowles DP (2008). Ovine herpesvirus 2 replicates initially in the lung of experimentally infected sheep. J Gen Virol.

[2] Cunha CW, Traul DL, Taus NS, Oaks JL, O’Toole D (2008). Detection of ovine herpesvirus 2 major capsid gene transcripts as an indicator of virus replication in shedding sheep and clinically affected animals. Virus Res.

[3] Schultheiss PC, Collins JK, Spraker TR, DeMartini JC (2000). Epizootic malignant catarrhal fever in three bison herds: differences from cattle and association with ovine herpesvirus-2. J Vet Diagn Invest.

[4] Løken T, Aleksandersen M, Reid H, Pow I (1998). Malignant catarrhal fever caused by ovine herpesvirus-2 in pigs in Norway. Vet Rec.

[5] Reid HW (1992). The Biology of Deer.

[6] Russell GC, Stewart JP, Haig DM (2009). Malignant catarrhal fever: a review. Vet J.

[7] O’Toole D, Li H (2014). The pathology of malignant catarrhal fever, with an emphasis on ovine herpesvirus 2. Vet Pathol.

[8] Taus NS, Schneider DA, Oaks JL, Yan H, Gailbreath KL (2010). Sheep (Ovis aries) airway epithelial cells support ovine herpesvirus 2 lytic replication in vivo. Vet Microbiol.

[9] Palmeira L, Sorel O, Van Campe W, Boudry C, Roels S (2013). An essential role for γ-herpesvirus latency-associated nuclear antigen homolog in an acute lymphoproliferative disease of cattle. Proc Natl Acad Sci USA.

[10] Sorel O, Chen T, Myster F, Javaux J, Vanderplasschen A (2017). Macavirus latency-associated protein evades immune detection through regulation of protein synthesis in cis depending upon its glycin/glutamate-rich domain. *PLoS Pathog*.

[11] Stewart M (2007). Molecular mechanism of the nuclear protein import cycle. Nat Rev Mol Cell Biol.

[12] Timney BL, Raveh B, Mironska R, Trivedi JM, Kim SJ (2016). Simple rules for passive diffusion through the nuclear pore complex. J Cell Biol.

[13] Görlich D, Kostka S, Kraft R, Dingwall C, Laskey RA (1995). Two different subunits of importin cooperate to recognize nuclear localization signals and bind them to the nuclear envelope. Curr Biol.

[14] O’Reilly AJ, Dacks JB, Field MC (2011). Evolution of the karyopherin-β family of nucleocytoplasmic transport factors; ancient origins and continued specialization. PLoS One.

[15] Wing CE, Fung HYJ, Chook YM (2022). Karyopherin-mediated nucleocytoplasmic transport. Nat Rev Mol Cell Biol.

[16] Soniat M, Chook YM (2015). Nuclear localization signals for four distinct karyopherin-β nuclear import systems. Biochem J.

[17] Kalderon D, Richardson WD, Markham AF, Smith AE (1984). Sequence requirements for nuclear location of simian virus 40 large-T antigen. Nature.

[18] Gualtiero A, Jans DA, Camozzi D, Avanzi S, Loregian A (2013). Regulated transport into the nucleus of herpesviridae DNA replication core proteins. Viruses.

[19] Cingolani G, Bednenko J, Gillespie MT, Gerace L (2002). Molecular basis for the recognition of a nonclassical nuclear localization signal by importin beta. Mol Cell.

[20] Fontes MR, Teh T, Kobe B (2000). Structural basis of recognition of monopartite and bipartite nuclear localization sequences by mammalian importin-alpha. J Mol Biol.

[21] Conti E, Uy M, Leighton L, Blobel G, Kuriyan J (1998). Crystallographic analysis of the recognition of a nuclear localization signal by the nuclear import factor karyopherin alpha. Cell.

[22] Kalita J, Kapinos LE, Lim RYH (2021). On the asymmetric partitioning of nucleocytoplasmic transport - recent insights and open questions. J Cell Sci.

[23] Görlich D, Panté N, Kutay U, Aebi U, Bischoff FR (1996). Identification of different roles for RanGDP and RanGTP in nuclear protein import. EMBO J.

[24] Jenner RG, Albà MM, Boshoff C, Kellam P (2001). Kaposi’s sarcoma-associated herpesvirus latent and lytic gene expression as revealed by DNA arrays. J Virol.

[25] Losay VA, Damania B (2025). Unraveling the kaposi sarcoma-associated herpesvirus (KSHV) lifecycle: an overview of latency, lytic replication, and KSHV-associated diseases. Viruses.

[26] Ballestas ME, Chatis PA, Kaye KM (1999). Efficient persistence of extrachromosomal KSHV DNA mediated by latency-associated nuclear antigen. *Science*.

[27] Cotter MA, Robertson ES (1999). The latency-associated nuclear antigen tethers the kaposi’s sarcoma-associated herpesvirus genome to host chromosomes in body cavity-based lymphoma cells. Virology.

[28] Cotter MA, Subramanian C, Robertson ES (2001). The kaposi’s sarcoma-associated herpesvirus latency-associated nuclear antigen binds to specific sequences at the left end of the viral genome through its carboxy-terminus. Virology.

[29] Garber AC, Hu J, Renne R (2002). Latency-associated nuclear antigen (LANA) cooperatively binds to two sites within the terminal repeat, and both sites contribute to the ability of LANA to suppress transcription and to facilitate DNA replication. J Biol Chem.

[30] Ballestas ME, Kaye KM (2001). Kaposi’s sarcoma-associated herpesvirus latency-associated nuclear antigen 1 mediates episome persistence through cis-acting terminal repeat (TR) sequence and specifically binds TR DNA. J Virol.

[31] Wong LY, Matchett GA, Wilson AC (2004). Transcriptional activation by the Kaposi’s sarcoma-associated herpesvirus latency-associated nuclear antigen is facilitated by an N-terminal chromatin-binding motif. J Virol.

[32] Renne R, Barry C, Dittmer D, Compitello N, Brown PO (2001). Modulation of cellular and viral gene expression by the latency-associated nuclear antigen of Kaposi’s sarcoma-associated herpesvirus. J Virol.

[33] Friborg J, Kong W, Hottiger MO, Nabel GJ (1999). p53 inhibition by the LANA protein of KSHV protects against cell death. Nature.

[34] Lu J, Jha HC, Verma SC, Sun Z, Banerjee S (2014). Kaposi’s sarcoma-associated herpesvirus-encoded LANA contributes to viral latent replication by activating phosphorylation of survivin. J Virol.

[35] Cherezova L, Burnside KL, Rose TM (2011). Conservation of complex nuclear localization signals utilizing classical and non-classical nuclear import pathways in LANA homologs of KSHV and RFHV. PLoS One.

[36] Piolot T, Tramier M, Coppey M, Nicolas JC, Marechal V (2001). Close but distinct regions of human herpesvirus 8 latency-associated nuclear antigen 1 are responsible for nuclear targeting and binding to human mitotic chromosomes. J Virol.

[37] Howard K, Cherezova L, DeMaster LK, Rose TM (2017). ORF73 LANA homologs of RRV and MneRV2 contain an extended RGG/RG-rich nuclear and nucleolar localization signal that interacts directly with importin β1 for non-classical nuclear import. Virology.

[38] Hart J, Ackermann M, Jayawardane G, Russell G, Haig DM (2007). Complete sequence and analysis of the ovine herpesvirus 2 genome. J Gen Virol.

[39] Kosugi S, Hasebe M, Tomita M, Yanagawa H (2009). Systematic identification of cell cycle-dependent yeast nucleocytoplasmic shuttling proteins by prediction of composite motifs. Proc Natl Acad Sci USA.

[40] Ariawan D, Thananthirige KPM, El-Omar A, van der Hoven J, Genoud S (2024). Design of peptide therapeutics as protein-protein interaction inhibitors to treat neurodegenerative diseases. RSC Adv.

[41] Munasinghe TS, Edwards MR, Tsimbalyuk S, Vogel OA, Smith KM (2022). MERS-CoV ORF4b employs an unusual binding mechanism to target IMPα and block innate immunity. Nat Commun.

[42] Teh T, Tiganis T, Kobe B (1999). Crystallization of importin α, the nuclear-import receptor. Acta Crystallogr D Biol Crystallogr.

[43] Studier FW (2005). Protein production by auto-induction in high density shaking cultures. Protein Expr Purif.

[44] Jagga B, Edwards M, Pagin M, Wagstaff KM, Aragão D (2021). Structural basis for nuclear import selectivity of pioneer transcription factor SOX2. Nat Commun.

[45] Aragão D, Aishima J, Cherukuvada H, Clarken R, Clift M (2018). MX2: a high-flux undulator microfocus beamline serving both the chemical and macromolecular crystallography communities at the Australian synchrotron. J Synchrotron Radiat.

[46] Battye TGG, Kontogiannis L, Johnson O, Powell HR, Leslie AGW (2011). iMOSFLM: a new graphical interface for diffraction-image processing with MOSFLM. Acta Crystallogr D Biol Crystallogr.

[47] Evans PR (2011). An introduction to data reduction: space-group determination, scaling and intensity statistics. Acta Crystallogr D Biol Crystallogr.

[48] Winn MD, Ballard CC, Cowtan KD, Dodson EJ, Emsley P (2011). Overview of the CCP4 suite and current developments. Acta Crystallogr D Biol Crystallogr.

[49] McCoy AJ, Grosse-Kunstleve RW, Adams PD, Winn MD, Storoni LC (2007). Phaser crystallographic software. JApCr.

[50] Emsley P, Lohkamp B, Scott WG, Cowtan K (2010). Features and development of coot. Acta Crystallographica Section D.

[51] Adams PD, Afonine PV, Bunkóczi G, Chen VB, Davis IW (2010). PHENIX: a comprehensive python-based system for macromolecular structure solution. Acta Crystallogr D Biol Crystallogr.

[52] Alvisi G, Manaresi E, Cross EM, Hoad M, Akbari N (2023). Importin α/β-dependent nuclear transport of human parvovirus B19 nonstructural protein 1 is essential for viral replication. Antiviral Res.

[53] Athukorala A, Donnelly CM, Pavan S, Nematollahzadeh S, Djossou VA (2024). Structural and functional characterization of siadenovirus core protein VII nuclear localization demonstrates the existence of multiple nuclear transport pathways. J Gen Virol.

[54] Cross EM, Akbari N, Ghassabian H, Hoad M, Pavan S (2024). A functional and structural comparative analysis of large tumor antigens reveals evolution of different importin α‐dependent nuclear localization signals. Protein Science.

[55] Cross EM, Marin O, Ariawan D, Aragao D, Cozza G (2023). Structural determinants of phosphorylation-dependent nuclear transport of HCMV DNA polymerase processivity factor UL44. FEBS Lett.

[56] Hoad M, Cross EM, Donnelly CM, Sarker S, Roby JA (2023). Structural characterization of porcine adeno-associated virus capsid protein with nuclear trafficking protein importin alpha reveals a bipartite nuclear localization signal. Viruses.

[57] Nematollahzadeh S, Athukorala A, Donnelly CM, Pavan S, Atelie-Djossou V (2024). Mechanistic insights into an ancient adenovirus precursor protein VII show multiple nuclear import receptor pathways. Traffic.

[58] Nath BK, Swarbrick CMD, Schwab RHM, Ariawan D, Tietz O (2025). Structural insights into the nuclear import of haliotid herpesvirus 1 large tegument protein homologue. Viruses.

[59] Smith KM, Di Antonio V, Bellucci L, Thomas DR, Caporuscio F (2018). Contribution of the residue at position 4 within classical nuclear localization signals to modulating interaction with importins and nuclear targeting. Biochim. Biophys. Acta - Mol. Cell Res.

[60] Alvisi G, Paolini L, Contarini A, Zambarda C, Di Antonio V (2018). Intersectin goes nuclear: secret life of an endocytic protein. Biochem J.

[61] Schindelin J, Arganda-Carreras I, Frise E, Kaynig V, Longair M (2012). Fiji: an open-source platform for biological-image analysis. Nat Methods.

[62] Lord SJ, Velle KB, Mullins RD, Fritz-Laylin LK (2020). SuperPlots: communicating reproducibility and variability in cell biology. J Cell Biol.

[63] Kosugi S, Hasebe M, Matsumura N, Takashima H, Miyamoto-Sato E (2009). Six classes of nuclear localization signals specific to different binding grooves of importin alpha. J Biol Chem.

[64] Hodel MR, Corbett AH, Hodel AE (2001). Dissection of a nuclear localization signal. J Biol Chem.

[65] Lange A, Mills RE, Lange CJ, Stewart M, Devine SE (2007). Classical nuclear localization signals: definition, function, and interaction with importin alpha. J Biol Chem.

[66] Nakada R, Hirano H, Matsuura Y (2017). Structural basis for the regulation of nuclear import of Epstein-Barr virus nuclear antigen 1 (EBNA1) by phosphorylation of the nuclear localization signal. Biochem Biophys Res Commun.

[67] Hoad M, Pavan S, Nematollahzadeh S, Tietz O, Reeman J (2026). Importin α/β1 dependent nuclear import of black sea bass polyomavirus large tumor antigen is mediated by a classical NLS located downstream of the SF3 helicase domain. Biochim Biophys Acta Mol Cell Res.

[68] Vogel OA, Forwood JK, Leung DW, Amarasinghe GK, Basler CF (2024). Viral Targeting of Importin Alpha-Mediated Nuclear Import to Block Innate Immunity. Cells.

[69] Marfori M, Lonhienne TG, Forwood JK, Kobe B (2012). Structural basis of high-affinity nuclear localization signal interactions with importin-α. Traffic.

[70] Wagstaff KM, Jans DA (2009). Importins and beyond: non-conventional nuclear transport mechanisms. Traffic.

[71] Henderson BR, Percipalle P (1997). Interactions between HIV Rev and nuclear import and export factors: the Rev nuclear localisation signal mediates specific binding to human importin-beta. J Mol Biol.

[72] Cros JF, García-Sastre A, Palese P (2005). An unconventional NLS is critical for the nuclear import of the influenza A virus nucleoprotein and ribonucleoprotein. Traffic.

[73] Dingwall C, Laskey RA (1991). Nuclear targeting sequences--a consensus?. Trends Biochem Sci.

[74] Conti E, Kuriyan J (2000). Crystallographic analysis of the specific yet versatile recognition of distinct nuclear localization signals by karyopherin alpha. Structure.

[75] Kobe B (1999). Autoinhibition by an internal nuclear localization signal revealed by the crystal structure of mammalian importin alpha. Nat Struct Biol.

[76] Bradley KJ, Bowl MR, Williams SE, Ahmad BN, Partridge CJ (2007). Parafibromin is a nuclear protein with a functional monopartite nuclear localization signal. Oncogene.

[77] Wang L, Li M, Cai M, Xing J, Wang S (2012). A PY-nuclear localization signal is required for nuclear accumulation of HCMV UL79 protein. Med Microbiol Immunol.

[78] Leemann-Zakaryan RP, Pahlich S, Grossenbacher D, Gehring H (2011). Tyrosine phosphorylation in the C-terminal nuclear localization and retention signal (C-NLS) of the EWS protein. Sarcoma.

[79] Schwam DR, Luciano RL, Mahajan SS, Wong L, Wilson AC (2000). Carboxy terminus of human herpesvirus 8 latency-associated nuclear antigen mediates dimerization, transcriptional repression, and targeting to nuclear bodies. J Virol.

[80] Stuber G, Mattsson K, Flaberg E, Kati E, Markasz L (2007). HHV-8 encoded LANA-1 alters the higher organization of the cell nucleus. Mol Cancer.

[81] Ballestas ME, Kaye KM (2011). The latency-associated nuclear antigen, a multifunctional protein central to Kaposi’s sarcoma-associated herpesvirus latency. Future Microbiol.

[82] Glenn M, Rainbow L, Auradé F, Davison A, Schulz TF (1999). Identification of a spliced gene from Kaposi’s sarcoma-associated herpesvirus encoding a protein with similarities to latent membrane proteins 1 and 2A of Epstein-Barr virus. J Virol.

