# Supplementary material for: Structural insights into the nuclear import of ovine gammaherpesvirus 2 ORF73 LANA homologue
Source: J Gen Virol. 2026 Apr 17;107(4):002250. doi: 10.1099/jgv.0.002250 (PMC13089313; doi:10.1099/jgv.0.002250)
Supplement: Fig. S1. [file jgv-107-02250-s001.pdf]

Structural insights into the nuclear import of ovine gammaherpesvirus 2 ORF73 LANA homolog

Babu Kanti Nath[1*], Renate H. M. Schwab[1], Crystall M. D. Swarbrick[1], Silvia Pavan[2], Nazia Rahman[3], Brian P. McSharry[1,4], Shane R. Raidal[5,6], Daryl Ariawan[7], Ole Tietz[7], Shubhagata Das[1,3,5], Gualtiero Alvisi[2] and Jade K. Forwood[1,3,5*]

[1]Gulbali Institute, Charles Sturt University, Wagga Wagga, NSW, Australia. bnath@csu.edu.au; rschwab@csu.edu.au; cswarbrick@csu.edu.au; bmcsharry@csu.edu.au; jforwood@csu.edu.au

[2]Department of Molecular Medicine, University of Padua, Padua, Italy. gualtiero.alvisi@unipd.it, silvia.pavan.1@unipd.it

[3]School of Agricultural, Environmental and Veterinary Sciences, Faculty of Science and Health, Charles Sturt University, Wagga Wagga, NSW, Australia. nrahman@csu.edu.au; jforwood@csu.edu.au; sdas@csu.edu.au

[4]School of Dentistry and Medical Sciences, Faculty of Science and Health, Charles Sturt University, Wagga Wagga, NSW, Australia. bmcsharry@csu.edu.au

[5]Training Hub Promoting Regional Industry and Innovation in Virology and Epidemiology, Gulbali Institute, Charles Sturt University, Wagga Wagga, NSW, Australia. jforwood@csu.edu.au; sdas@csu.edu.au; shane.raidal@unimelb.edu.au

[6]Melbourne Veterinary School, Faculty of Science, University of Melbourne, Victoria, Australia. shane.raidal@unimelb.edu.au

[7]Dementia Research Centre, Macquarie Medical School, Faculty of Medicine, Health and Human Sciences, Macquarie University, North Ryde, Sydney, NSW 2109, Australia. ole.tietz@mq.edu.au; daryl.ariawan@mq.edu.au

* = Correspondence: Babu Nath, bnath@csu.edu.au

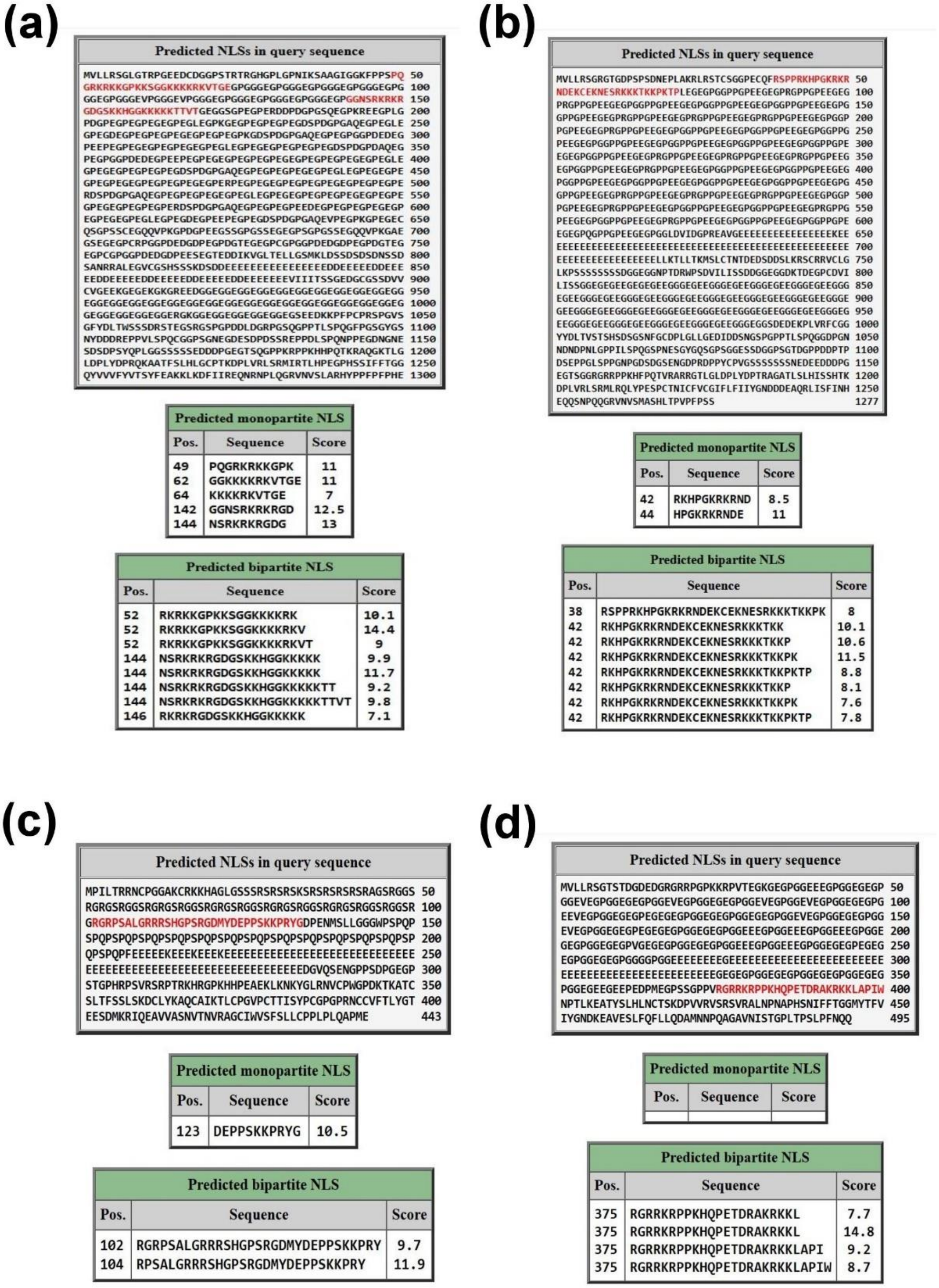

Supplementary Figure 1: cNLS mapper results. LANA amino acid sequence of four representative virus of macavirus genus (a) Alcelaphine herpesvirus 1 (AlGHV1), (b) Alcelaphine herpesvirus 2 (AlGHV2) (c) Bovine gammaherpesvirus 6 (BoGHV6) and (d) Ovine gammaherpesvirus 2 (OvGHV2) was retrieved from GeneBank and analysed with cNLS mapper for identification of putative cNLSs. Top panel: The protein sequence is displayed using the single letter amino acid code, with identified putative cNLSs shown in red. Bottom panels: The predicted cNLS sequences are shown, along with the position of the first amino acid and the predicted cNLS mapper score.

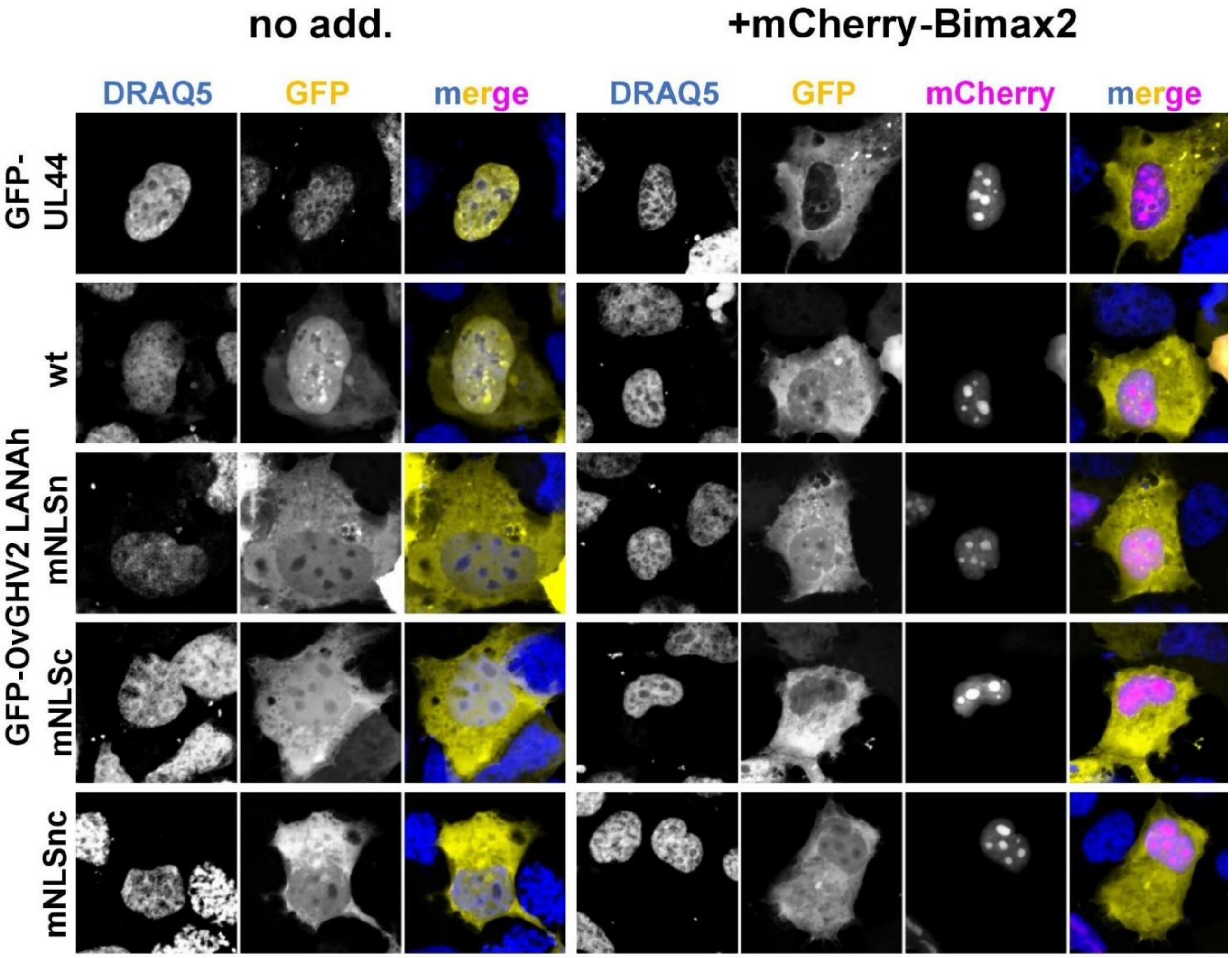

Supplementary Figure 2: Individual channels for micrographs shown in Figure 6B.

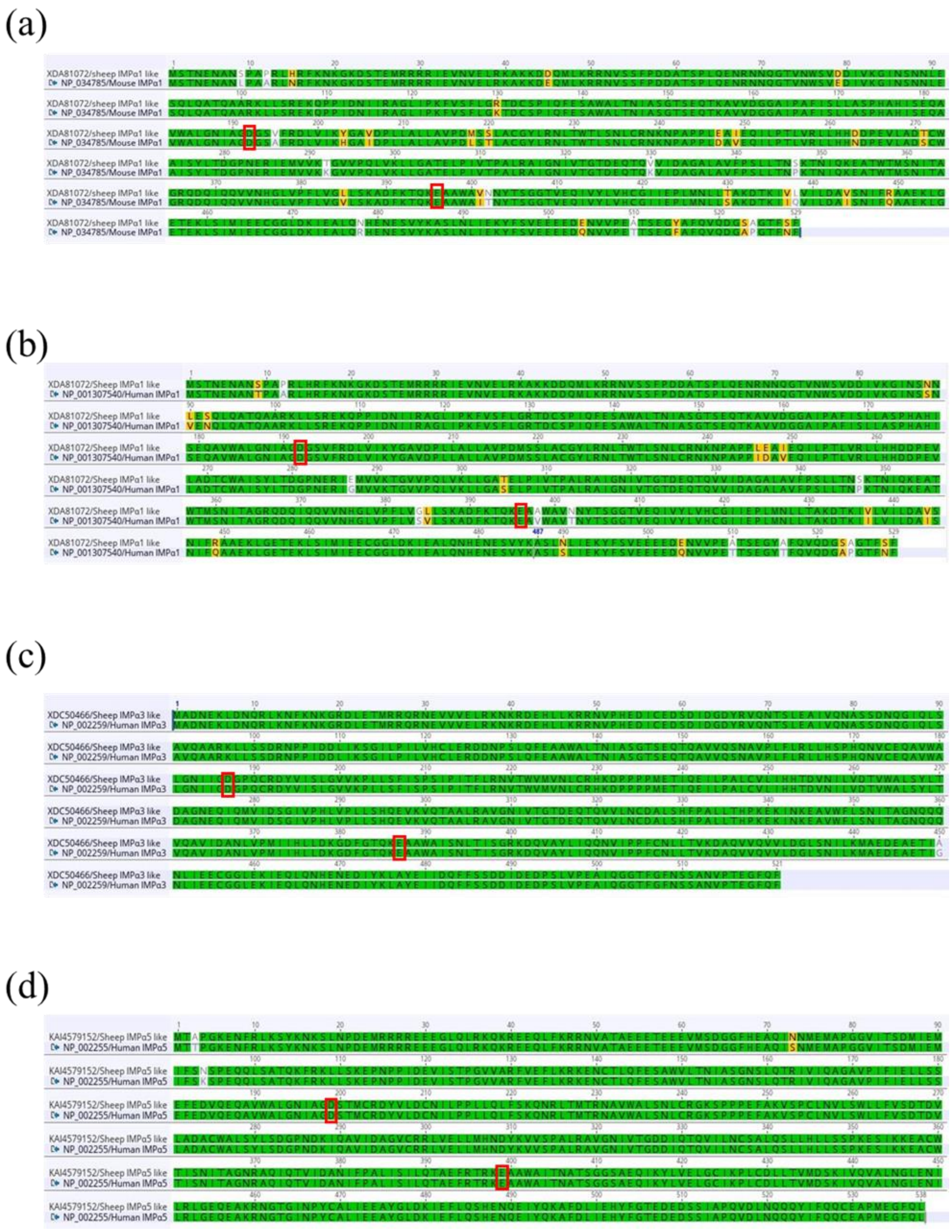

Supplementary Figure 3: Alignment of sheep and human/mouse IMPα amino acid sequences. (a) Mouse IMPα1 (named as IMPα2) and sheep IMPα1-like (93.76% homology) (b) Human IMPα1 and sheep IMPα1-like (95.27% homology) (c) Human IMPα3 and sheep IMPα3-like (99.81% homology) (d) Human IMPα5 and sheep IMPα5-like (99.44% homology). Alignment was performed using the MAFFT L-INS-I algorithm within

Geneious Prime (version 7.388). Conserved major (P2) and minor (P2´) binding sites of human and sheep IMPα are highlighted in the red box within the alignment.

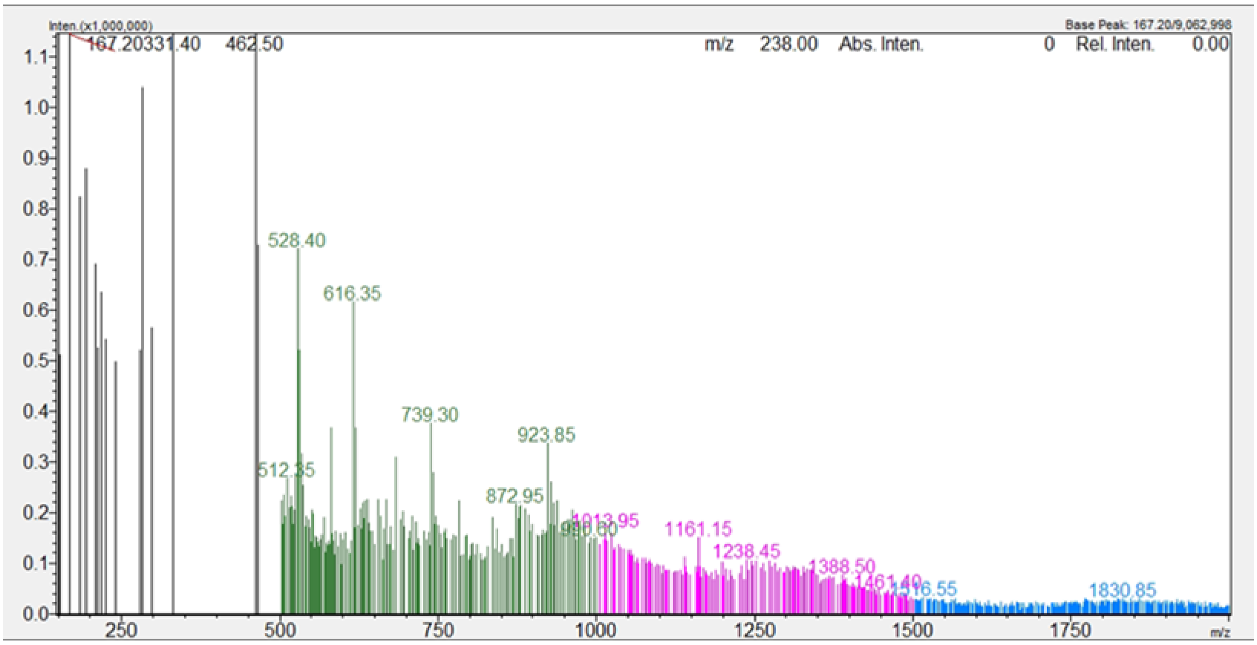

HPLC trace

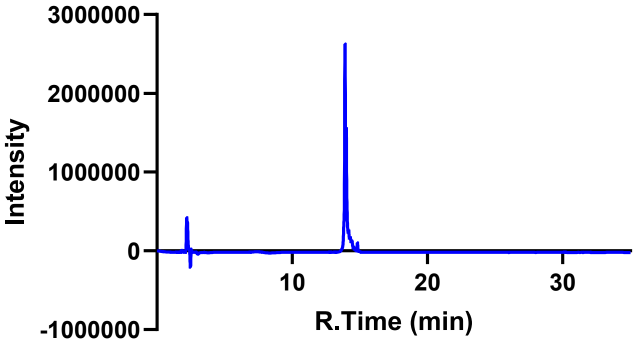

Peak table

Peak#	R.Time	I.Time	F.Time	Area	Area %
1	13.168	13.125	13.173	1128	0.007309
2	13.344	13.253	13.349	1429	0.009259
**3**	**14.172**	**13.253**	**14.187**	**15431022**	**99.98343**
Total				15433579.00	100

Supplementary Figure 4: Peptide synthesis of NLS. (a)Molecular Weight: 3708.43 g mol-1; LCMS [ESI+]: 923.85 (m/z4; calculated: 923.51), 739.30 (m/z5; calculated: 739.01), 616.35 (m/z6; calculated: 616.01), 528.40 (m/z5; calculated: 528.15) [Found as water loss, -18 total mass]. (b) HPLC spectra and the peak table to analyse the purity.HPLC retention time rt = 13.8 min, Purity = 99 %. The AUC was analysed using the built-in software from Shimadzu HPLC. The peak at ~2 min is not included as this peak resulted from the pressure of the sample injection.

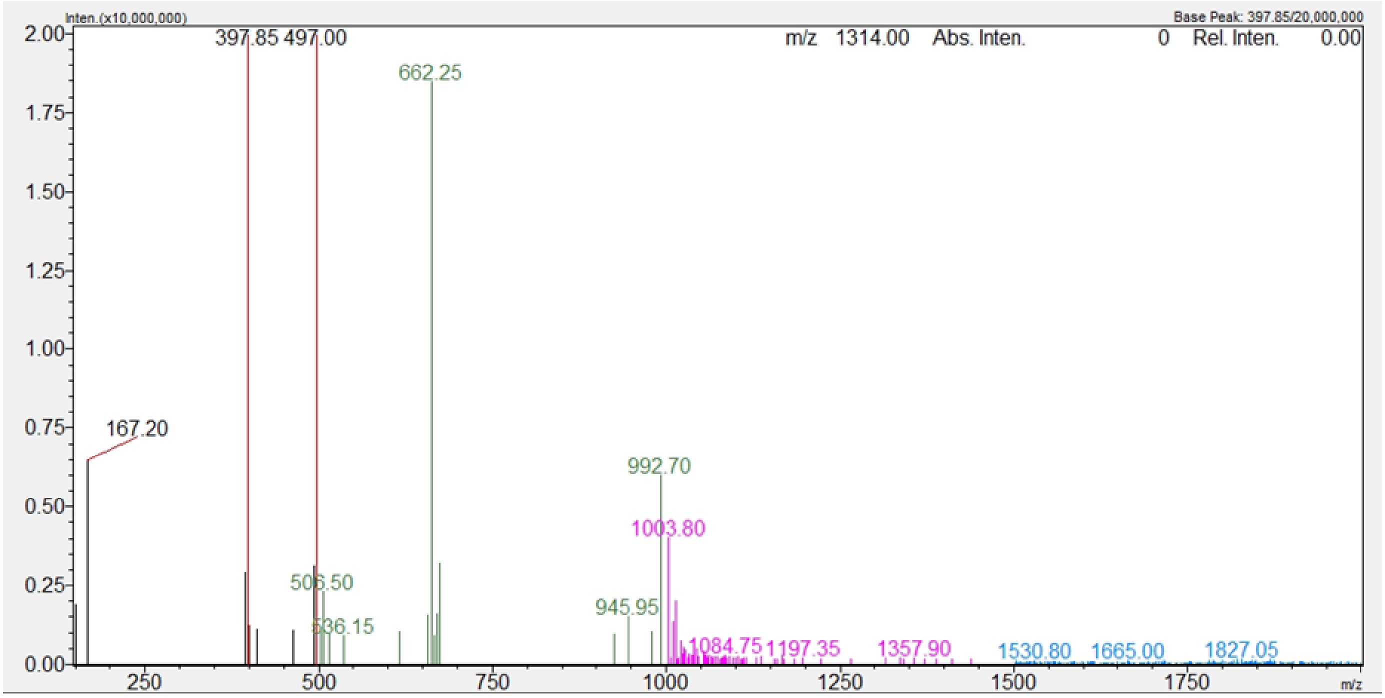

HPLC trace

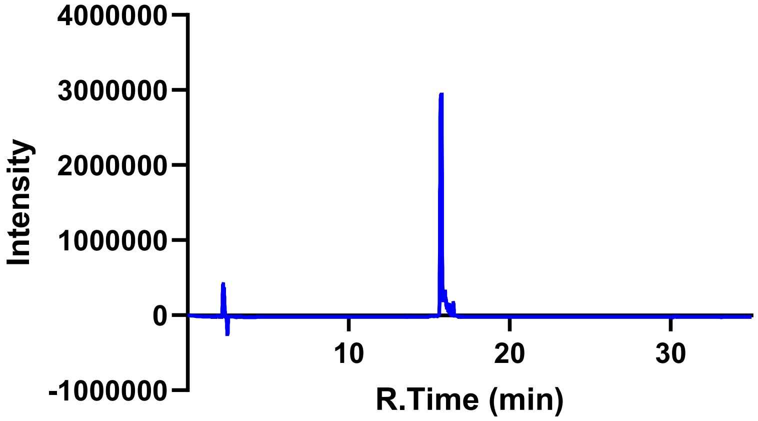

Peak table

Peak#	R.Time	I.Time	F.Time	Area	Area %
1	15.372	15.328	15.44	15652	0.05459
**2**	**15.773**	**15.515**	**16.203**	**27610177**	**96.29644**
3	16.245	16.203	16.315	420765	1.467509
4	16.5	16.432	16.56	625469	2.181458
Total				28672063.00	100

Supplementary Figure 5: Peptide synthesis of NLSΔ375-388. (a) Molecular Weight: 1983.43 g mol-1; LCMS [ESI+]: 992.70 (m/z_2; calculated: 992.72), 662.25 (m/z_3; calculated: 662.15), 497.00 (m/z_3; calculated: 496.87), 397.85 (m/z_3; calculated: 397.69). (b) HPLC spectra and the peak table to analyse the purity. HPLC retention time r_t = 15.6 min; Purity = 96 %. The AUC was analysed using the built-in software from Shimadzu HPLC. The peak at ~2 min is not included as this peak resulted from the pressure of the sample injection.

Supplementary Table 1: Amino acids sequence similarities of LANA homolog from the selected Gammaherpesviruses

		1	2	3	4	5	6	7	8	9	10	11	12	13	14	15	16	17	18	19	20	21	22
1	AAC58118/alcelaphine gammaherpesvirus 1	100																					
2	AAX58107/ovine gammaherpesvirus 2	23.14																					
3	AIA62108/alcelaphine gammaherpesvirus 2	45.63	27.75																				
4	AAC95598/ateline gammaherpesvirus 3	6.62	11.52	7.03																			
5	CAA45696/saimiriine gammaherpesvirus 2	7.93	17	9	30.26																		
6	AF148805/human gammaherpesvirus 8	11.78	10.05	12.9	5.51	12.08																	
7	AGY30760/macacine gammaherpesvirus 8	15.97	12.85	17.48	5.64	12.5	29.54																
8	AGY30760/macacine gammaherpesvirus 12	15.97	12.85	17.48	5.64	12.5	29.54	100															
9	AIU39597/equid gammaherpesvirus 5	14.53	11.7	17.83	6.41	6.33	8.96	23.15	23.15														
10	AJF36130/equid gammaherpesvirus 2	13.13	8.81	15.53	5.02	6.88	9.95	23.92	23.92	45.92													
11	ALE14789/felid gammaherpesvirus 1	9.46	15.56	10.68	14.85	19.41	10.95	13.74	13.74	13.34	12.04												
12	AIB03226/bovine gammaherpesvirus 6	9.95	16.77	10.17	7.97	14.73	10.04	11.12	11.12	8.38	8.85	20.03											
13	ARW78134/delphinid gammaherpesvirus 1	6.24	7.44	6.92	6.53	6.27	6.28	8.13	8.13	12.5	11.84	11.72	13.04										
14	AAK95440/macacine gammaherpesvirus 4	7.15	10.72	7.84	9.24	7.92	5	4.51	4.51	7.16	5.57	8.69	9.49	8.21									
15	ALF03247/macacine gammaherpesvirus 10	7	11.01	8.06	9.53	8.1	5	4.42	4.42	7.16	5.66	9	9.62	8.02	89.04								
16	CAD53427/human gammaherpesvirus 4	9.61	15.29	11.41	7.93	7.6	5.33	5.25	5.25	10.85	7.63	9.31	8.72	8.25	41.48	41.59							
17	AF319782/callitrichine gammaherpesvirus 3	3.94	7.5	4.6	12.14	9.33	3.98	4.19	4.19	5.78	5.41	11.83	8.91	7.92	26.35	24.76	20.12						
18	AAD21406/macacine gammaherpesvirus 5	6.83	8.52	7.15	10.57	10.62	8.53	10.37	10.37	10.45	10.04	13.92	15.05	14.43	10.45	10.24	8.66	11.9					
19	AAT00130/macacine gammaherpesvirus 11	5.98	7.75	6.37	5.97	5.56	5.42	7.21	7.21	8.21	8.01	9.89	13.13	12.08	6.99	6.79	4.93	6.38	67.73				
20	ACY41142/murid gammaherpesvirus 7	3.77	4.61	3.68	7.36	7.8	6.5	6.95	6.95	6.73	8.04	12.15	9.25	10.99	5.51	5.49	5.68	9.62	17.56	12.2			
21	ADW24414/cricetid gammaherpesvirus 2	3.73	5.24	4.02	7.66	9.09	4.57	5.88	5.88	5.74	6.46	10.13	9.57	10.2	5.64	5.81	5.55	9.9	15.29	10.13	27.56		
22	ATA58303/vespertilionid gammaherpesvirus 3	8.02	6.87	8.07	6.48	7.63	9.53	10.79	10.79	11.64	11.15	9.07	6.35	6.59	6.01	6.2	8.2	5.34	9.73	7.48	5.6	5.99	

Supplementary Table 2: LANA amino acid sequence length, predicted NLS position and type of diverse Gammaherepsviruses

Name of the virus and accession number	Length of amino acid	Position of NLS	Type of NLS
AAC58118/alcelaphine gammaherpesvirus 1	1300	N	Both
AAC95598/ateline gammaherpesvirus 3	447	N	Monopartite
AAD21406/macacine gammaherpesvirus 5	448	NP	NP
AAK95440/macacine gammaherpesvirus 4	511	NP	NP
AAT00130/macacine gammaherpesvirus 11	436	NP	NP
AAX58107/ovine gammaherpesvirus 2	495	C	Bipartite
ACY41142/murid gammaherpesvirus 7	327	N	Both
ADW24414/cricetid gammaherpesvirus 2	294	NP	NP
AF148805/human gammaherpesvirus 8	1129	N	Both
AF319782/callitrichine gammaherpesvirus 3	327	NP	NP
AGY30760/macacine gammaherpesvirus 8	1071	N	Both
AGY30760/macacine gammaherpesvirus 12	1071	N	Both
AIA62108/alcelaphine gammaherpesvirus 2	1277	N	Both
AIB03226/bovine gammaherpesvirus 6	443	N	Both
AIU39597/equid gammaherpesvirus 5	996	N	Both
AJF36130/equid gammaherpesvirus 2	985	N and C	Both
ALE14789/felid gammaherpesvirus 1	518	N and Internal	Both
ALF03247/macacine gammaherpesvirus 10	519	N	Mono
ARW78134/delphinid gammaherpesvirus 1	513	N and C	Both
ATA58303/vespertilionid gammaherpesvirus 3	1010	Internal	Mono
CAA45696/saimiriine gammaherpesvirus 2	407	N	Mono
CAD53427/human gammaherpesvirus 4	641	N and C	Bipartite

N=N-terminal; C=C-terminal, NP= Not predicted; Both= Monopartite and bipartite
